# Neuronal transcription of autism gene *PTCHD1* is regulated by a conserved downstream enhancer sequence

**DOI:** 10.1038/s41598-023-46673-0

**Published:** 2023-11-21

**Authors:** Stephen F. Pastore, Tahir Muhammad, Cassandra Stan, Paul W. Frankland, Paul A. Hamel, John B. Vincent

**Affiliations:** 1 Molecular Neuropsychiatry & Development (MiND) Lab, Molecular Brain Science Research Department, Campbell Family Mental Health Research Institute, Centre for Addiction and Mental Health, Toronto, ON M5T 1RS Canada; 2 Institute of Medical Science, University of Toronto, Toronto, ON M5S 1A8 Canada; 3 Program in Neurosciences and Mental Health, The Hospital for Sick Children, Toronto, ON M5G 1X8 Canada; 4 Department of Physiology, University of Toronto, Toronto, ON M5S 1A8 Canada; 5 Department of Psychology, University of Toronto, Toronto, ON M5S 3G3 Canada; 6 Department of Laboratory Medicine & Pathobiology, University of Toronto, Toronto, ON M5S 1A8 Canada; 7 Department of Psychiatry, University of Toronto, Toronto, ON M5T 1R8 Canada; 8 Molecular Brain Science Research Department, Campbell Family Mental Health Research Institute, Centre for Addiction and Mental Health (CAMH), 250 College Street, Toronto, ON M5T 1R8 Canada

**Keywords:** Gene expression, Gene regulation, Neurodevelopmental disorders

## Abstract

Patched domain-containing 1 (*PTCHD1*) is a well-established susceptibility gene for autism spectrum disorder (ASD) and intellectual disability (ID). Previous studies have suggested that alterations in the dosage of *PTCHD1* may contribute to the etiology of both ASD and ID. However, there has not yet been a thorough investigation regarding mechanisms that regulate *PTCHD1* expression. We sought to characterize the *Ptchd1* promoter in a mouse neuronal model, as well as to identify and validate *cis* regulatory elements. We defined specific regions of the *Ptchd1* promoter essential for robust expression in P19-induced neurons. Evolutionarily-conserved putative transcription factor binding sites within these regions were subsequently identified. Using a pairwise comparison of chromatin accessibility between mouse forebrain and liver tissues, a candidate regulatory region, ~ 9.1 kbp downstream of the *Ptchd1* stop codon was defined. This region harbours two ENCODE-predicted enhancer *cis*-regulatory elements. Further, using DNase footprint analysis, a putative YY1-binding motif was also identified. Genomic deletion of the entire 8 kbp downstream open chromatin region attenuated *Ptchd1* transcription by over 60% in our neuronal model, corroborating its predicted regulatory function. This study provides mechanistic insights related to the expression of *PTCHD1*, and provides important context to interpret genetic and genomic variation at this locus which may influence neurodevelopment.

## Introduction

Autism spectrum disorder (ASD) is a heterogeneous neurodevelopmental condition that is characterized by circumscribed and repetitive sensory-motor behaviours, as well as impairments in social communication^1^. These features along this spectrum range from relatively mild to severely debilitating. The prevalence of ASD in developed countries has recently been estimated to be approximately 1.5%^2^, with a three-fold higher incidence in males^3^. The pathoetiology of ASD has a substantial genetic component. A recent meta-analysis of twin studies suggested that 74–93% of ASD risk is heritable^4^. Concomitantly, studies of ASD on a genome-wide scale have identified a multitude of ASD and intellectual disability (ID) susceptibility genes.

One specific risk factor gene for ASD and ID is Patched domain-containing 1 (*PTCHD1*) (MIM: 300828)^5^. Studies have further implicated *PTCHD1* as an ASD and ID susceptibility gene by identifying additional rare genomic *PTCHD1* variants, including both microdeletion copy number variants (CNVs) and single-nucleotide variants (SNVs)^6^. The function of PTCHD1 protein has yet to be comprehensively elucidated, although in silico analyses predict it to be a multi-pass transmembrane protein with two putative ligand-binding exoplasmic loops, sterol-sensing domains and a C-terminal PDZ-binding domain^6^. Although studies suggest that PTCHD1, unlike PTCH1, does not play a role in the Sonic Hedgehog (SHH)/Smoothened (SMO) signaling pathway^7,8^, PTCHD1 has been shown to bind to cholesterol in vitro^9^.

*PTCHD1* is encoded on the X-chromosome at the locus Xp22.11 in humans, and *Ptchd1* on XqF3 in mice. During embryonic and postnatal development in mice, *Ptchd1* has both variable temporal and brain subregion-specific levels of transcription^7,8,10–12^, suggesting that a coordinated spatial synchrony of *PTCHD1* expression may be essential for proper neurodevelopment. It has also been observed that depolarization of mouse primary cortical neurons^13^ and human induced pluripotent stem cell-derived neurons yielded significant increases in the expression of *Ptchd1* and *PTCHD1*, respectively^14^, consistent with an activity-dependent role in mature neurons.

In addition to CNVs, potential expression-altering variants at the *PTCHD1* locus that do not affect its coding sequence (CDS) have also been linked to ASD and ID. The single-nucleotide polymorphism (SNP) rs7052177 is reported to be associated with ASD and ID^15^. This SNP is situated in the first intron of *PTCHD1* within a putative binding site for the members of the signal transducer and activator of transcription (STAT) family of transcription factors STAT3, STAT5A, and STAT5B, which are all predicted to bind preferentially to the rs7052177T allele^15^ and therefore may conceivably affect the expression of *PTCHD1*. Moreover, the authors also identified a 27 bp duplication within the *PTCHD1* promoter in three individuals with ASD that correlated with a 26% reduction in transcription in vitro^15^. Lastly, the longest allele of a microsatellite, consisting of 14 repeats of a [GCC] trinucleotide repeat, was found to be associated with ASD^15^. Collectively, these data suggest that the dosage of *PTCHD1* may be an important variable that requires precise control during neurodevelopment.

While it has been postulated that *PTCHD1* may be a dosage-sensitive gene with implications for ASD and ID^15^, there have been no comprehensive examinations regarding the mechanisms that influence its expression. In this study, we have defined specific segments of the proximal region upstream of that regulate its transcription. Further, we identified and validated a putative regulatory region that is predicted to contain enhancer elements located downstream of *Ptchd1*.

## Materials and methods

### Plasmids

In order to facilitate the generation of P19 cell lines stably expressing reporter constructs containing *Ptchd1* promoter truncations, a secondary cassette encoding puromycin resistance was cloned and inserted between the f1 origin of replication and the synthetic polyadenylation signal of the luciferase reporter vector pBV-Luc (addgene.org; Fig. S1). Segments of the proximal genomic region upstream of *Ptchd1* spanning from − 1782 to + 17 relative to the annotated transcription start site (TSS) (chrX: 154,406,321; GRCm39) were ligated into the multiple cloning site of the modified vector (Fig. 2A). To prevent recombination-mediated disruption of either gene cassette during stable cell line generation, all plasmids were linearized and agarose gel-purified prior to transfection. Cloning primer sequences are provided in Table S1.

### Cell culture

The model system for this study is the male mouse-derived pluripotent embryonal carcinoma cell line P19^16^. These well-characterized cells can be reliably differentiated into enriched neuronal cells, and provide a useful model to study neuronal differentiation in vitro^17–20^. These cells were maintained in α-MEM supplemented with 10% fetal bovine serum (FBS) (Wisent; Saint-Jean-Baptiste, QC, Canada) and 1% penicillin–streptomycin (PS) (Wisent) in T75 flasks (Sarstedt; Nümbrecht, Germany). Cells were passaged every 48–72 h at semi-confluence by trypsinization (Wisent).

### Stable cell line generation

To generate stable cell lines expressing *Ptchd1* reporter constructs, cells were first re-seeded in 6-well culture plates (Falcon; Corning, NY). 24 h later, Lipofectamine 3000 (Thermo Fisher Scientific; Waltham, MA) was utilized according to the manufacturer’s instructions to first transfect linearized plasmids into P19 cells. 24 h post-transfection, cells were trypsinized, re-seeded in T75 flasks containing α-MEM supplemented with 1 μg/mL puromycin (BioShop; Burlington, ON, Canada) and cultured for 48–72 h. Stably-transfected cells were passaged 2–3 additional times in puromycin-supplemented α-MEM before being either used for the induction of differentiation or frozen for long-term storage. In order to eliminate potential bias through inter-clone variability, genomic location and number of sites of integration of the reporter construct, we used a polyclonal approach, rather than selecting and amplifying single transfected clones (monoclonal).

### Differentiation of P19 cells

Differentiation of P19 cells was carried out as described previously^20^ with minor modifications. Briefly, cells were trypsinized, resuspended in neural induction medium [α-MEM supplemented with 2.5% FBS, 1% PS, 1X GlutaMAX (Thermo Fisher Scientific, Waltham, MA) and 1 μM all-trans retinoic acid (RA) (Cell Signaling; Danvers, MA)] and 6 × 10^4^ cells per cm^2^ were seeded in T75 flasks for four days, with a medium change after the second day. Following induction, cells were trypsinized, resuspended in neural growth medium I [Neurocell medium (Wisent) supplemented with 1X GlutaMAX and 1X N2 supplement (Wisent)] and 9 × 10^4^ cells per cm^2^ were seeded in 6-well plates coated with Matrigel (Corning) [this time point is hereafter referred to as days after induction (DAI) 0] for five days, with a medium change after 2–3 days. On DAI 5, the medium was replaced with neural differentiation medium II [Neurocell medium supplemented with 1X GlutaMAX, 1X W21 supplement (Wisent), 8 μM cytosine arabinoside (AraC) (Sigma-Aldrich; St. Louis, MO) and 8 nM 2′-deoxycytidine (Thermo Fisher Scientific, Waltham, MA)] for five days, with a medium change after 2–3 days. On DAI 10, the medium was replaced with neural differentiation medium III (Neurocell medium supplemented with 1X GlutaMAX and 1X W21 supplement) for up to 10 days, with medium changes every 2–3 days.

### Neuronal depolarization

To evaluate activity-dependent induction of *Ptchd1* in our model, neurons were depolarized at DAI20 by incubation with differentiation medium III containing 55 mM potassium chloride for 6 h, followed by immediate cell lysis and downstream experimentation. For non-depolarized control neurons, fresh differentiation medium III without potassium chloride was added for the same duration.

### RNA isolation and RT-qPCR

To isolate RNA from cells, the NucleoSpin RNA Mini Kit (Machery-Nagel; Düren, Germany) was utilized according to the manufacturer’s instructions, with cells being lysed directly in the 6-well culture plate following a PBS wash. Following isolation, RNA integrity was verified by agarose gel electrophoresis. To generate cDNA, 1 μg of RNA was reverse-transcribed using the iScript cDNA Synthesis Kit (Bio-Rad; Hercules, CA).

For qPCR, reactions were prepared using the PowerUp SYBR Green Master Mix (Thermo Fisher Scientific, Waltham, MA), and template cDNA was diluted between two- and 1000-fold in order to ensure that amplification was occurring within the linear dynamic range for the given primer pair. All qPCR primer sequences and reaction conditions are specified in Table S2. For each biological replicate, four technical replicates were prepared. Reactions were amplified in 384-well plates using the QuantStudio Real-Time PCR System software on the ViiA7 System instrument (Thermo Fisher Scientific, Waltham, MA) with the ΔΔCt experimental setting and standard amplification properties. Each reaction was subjected to a melt curve analysis, and all instrument runs included a non-template control for each primer pair. For biological replicates from each target gene, Ct values from all technical replicates were averaged, and Ct values from control biological replicates were further averaged (Ct-control). Ct values from experimental biological replicates were subtracted from Ct-control [ΔCP(control-experimental)]. Fold-changes for target genes were calculated by taking the ratio between E_target_^ΔCP(control-experimental)^ and E_housekeeping_^ΔCP(control-experimental)^, with *E* being the efficiency of each primer set^21^ (Table S2). For normalization, ΔCP values were averaged for the two housekeeping genes *Gapdh* and *β-actin*.

### Luciferase activity assay

To evaluate luciferase activity, the Luciferase Assay System (Promega; Madison, WI) was used according to the manufacturer’s instructions, with the 1X Reporter Lysis Buffer being used to lyse neurons directly in the 6-well culture plate at DAI 16. This terminal time point was selected because, in contrast with the parental P19 line, neurons derived from P19 clones were observed to exhibit pronounced cell death after DAI 17. Bioluminescence was quantified using the Wallac 1420 software on the VICTOR3 Multilabel Plate Reader instrument (PerkinElmer; Waltham, MA). For each biological replicate, bioluminescence values for technical replicates were averaged and normalized to total protein, which was quantified from the Bradford protein assay.

### Comparative evolutionary genomic analyses and sequence alignments

To estimate the evolutionary conservation of the 1.8 kbp region upstream of the *PTCHD1* CDS, sequence comparisons were performed between mouse, rat, macaque and human using ECR Browser^22^, with evolutionary conserved regions (ECRs) recognized using a similarity percentage of 70%. Sequence alignments were synthesized by ClustalX2^23^.

### Transcription factor binding site predictions

Putative transcription factor binding sites (TFBSs) within the *PTCHD1* promoter were inferred using the SwissRegulon database^24^ (expasy.org/resources/swissregulon). Similar to Guaraldo et al., candidate TFBSs were selected if they exhibited sufficient sequence conservation between the mouse, macaque and human genomes^25^, and possessed position-frequency matrix scores of greater than 0.8.

### DNase I hypersensitive site analysis, footprint identification and motif discovery

To identify and characterize prospective regulatory elements proximal or distal to *Ptchd1*, comparative computational analyses were conducted on publicly-accessible next-generation sequencing data. For these analyses, we chose to compare DNase-seq and ChIP-seq data from mouse postnatal day 0 (P0) tissues that exhibited relatively high forebrain and low liver levels of *PTCHD1* expression^6^. We downloaded the call sets uploaded by Prof. Stamatoyannopoulos (University of Washington) from the ENCODE portal^26^ (encodeproject.org/) with the following identifiers: ENCSR791AJY (forebrain; DNase-seq), ENCSR258YWW (forebrain; H3K4me3 ChIP-seq), ENCSR094TTT (forebrain; H3K27ac ChIP-seq), ENCSR216UMD (liver; DNase-seq), ENCSR653AVN (liver; H3K4me3 ChIP-seq), and ENCSR616TJM (liver; H3K27ac ChIP-seq). We set the range for the screen at a ~ 406 kbp window (chrX: 154,181,675–154,587,778; GRCm39), including ~ 181 kbp sequence upstream and ~ 171 kbp downstream of the mouse *Ptchd1-a* transcript (NM_001093750.1). No other known protein coding genes lie within this region, or within the syntenic region on the human X chromosome. Additional comparisons in chromatin accessibility were made between human embryonic brain (ENCSR649KBB) and liver (ENCSR562FNN), between mouse P0 forebrain with midbrain (ENCSR767AJS) and hindbrain (ENCSR469VGZ), and between mouse P0 forebrain with liver, heart, stomach, kidney, and small intestine.

For DNase footprints, we used the Wellington algorithm from the pyDNase package^27^. Following DNase footprint identification, motif discovery was conducted using the HOMER suite of tools^28^.

### CRISPR-Cas9 deletion of downstream open chromatin region

To assess the contribution of the downstream open chromatin region on *Ptchd1* transcription, the entire genomic region was deleted by CRISPR-Cas9. Two 20 bp protospacer sequences upstream of NGG protospacer-adjacent motifs (PAM) that flank the downstream accessible region to be deleted (chrX: 154,339,382–154,347,408; GRCm39) were identified (Fig. 6A). Candidate protospacers were screened to mitigate off-target effects using Off-Spotter^29^. To generate the two single guide RNAs (sgRNAs), oligonucleotides comprising the protospacers were inserted into the linearized vector pX459 (www.addgene.org). In order to delete the downstream accessible region, P19 cells were transfected in 6-well plates with both sgRNA-encoding Cas9 vectors. 24 h post-transfection, cells were trypsinized, re-seeded in T75 flasks containing α-MEM supplemented with 1 μg/mL puromycin, and cultured for 48–72 h. Cells were then re-seeded at low density in 96-well plates (Falcon) and cultured for seven days, at which point monoclonal wells were identified and subsequently expanded. Positive clones were first screened for the correct deletion by PCR, and the deletion was subsequently confirmed by Sanger sequencing (Fig. 6C). Confirmed positive clones were passaged 2–3 additional times before being either used for the induction of differentiation or frozen for long-term storage. For comparative analyses, non-deletion clones were generated in the same manner using only one of the sgRNA-encoding Cas9 vectors. The genomic regions surrounding the sgRNA1 and sgRNA2 protospacers were sequenced for all candidate control lines. Following transfection and puromycin selection, most control clones selected for this study exhibited no genomic changes, and two clones contained very small 1–7 bp indels within the protospacer. In the latter case, such small indels relative to the size of the 8 kbp deletion region are unlikely to have altered chromatin modification and accessibility. Protospacers and screening primers are listed in Table S3.

## Results

### Induction of *Ptchd1* in differentiated P19 cells

**Figure 1:**
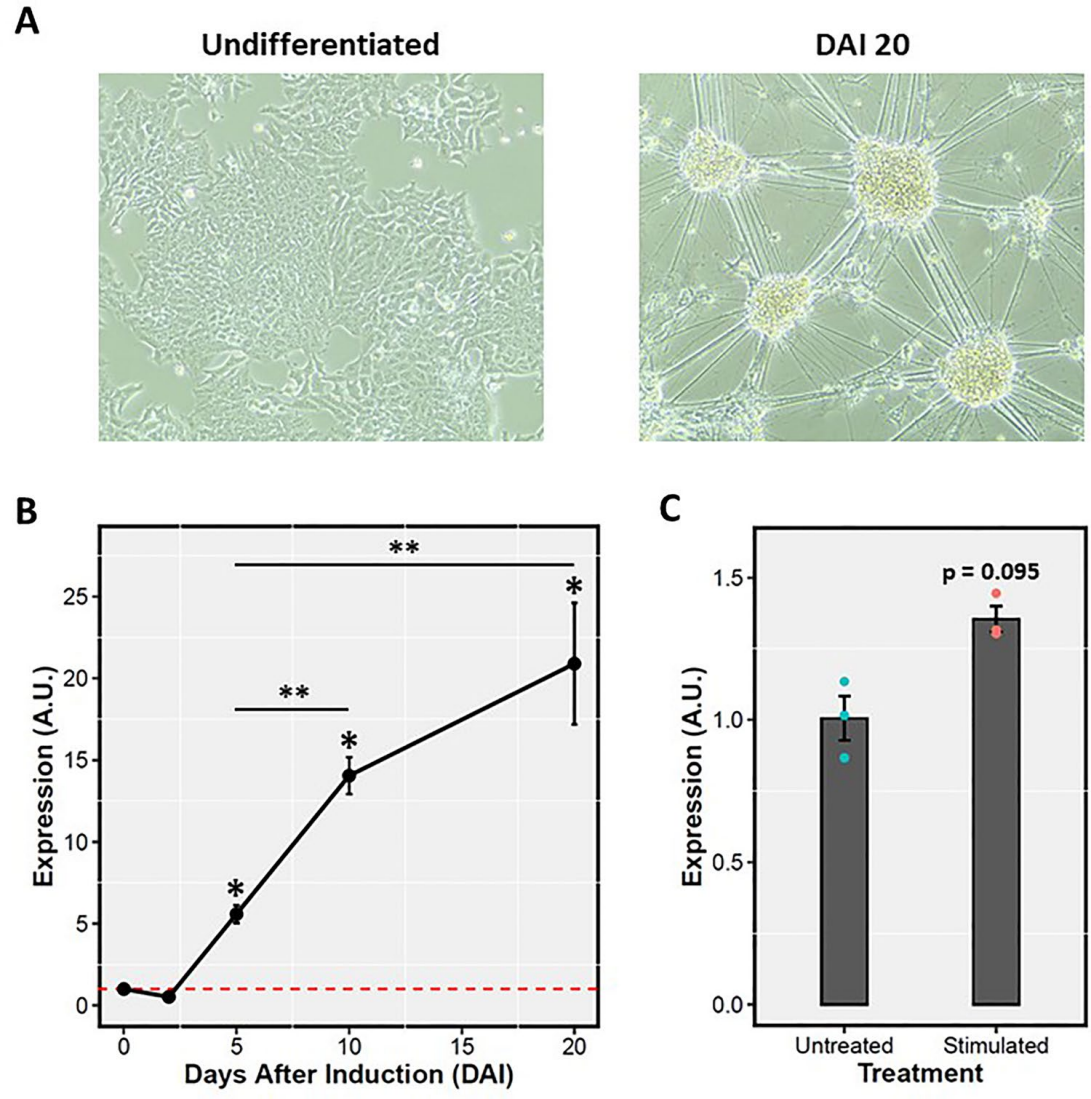
Induction of *Ptchd1* with neural differentiation in P19 cells. (**A**) Bright field microscope images (10 × magnification) depicting undifferentiated (*left image*) and DAI 20 (*right image*) P19 cells. (**B**) *Ptchd1-a* mRNA levels at DAI 0, 2, 5, 10 and 20, expressed as fold-changes relative to DAI 0. Data are expressed as the mean ± SEM for each group and were analyzed using a one-way ANOVA followed by a Tukey’s HSD test (*p ≤ 0.001 relative to DAI 0, **p ≤ 0.0001 between the indicated groups; n = 3 independent biological replicates per time point). (**C**) *Ptchd1-a* mRNA levels at DAI 20 basally and after depolarization with 55 mM KCl for 6 h, expressed as a fold-change relative to the untreated neurons. Data are expressed as the mean ± SEM for each group and were analyzed using a student’s unpaired t-test (n = 3 independent biological replicates per group).

To assess the *Ptchd1-a* expression profile of P19 cells during differentiation up to DAI 20 (Fig. 1A), RNA was isolated at DAI 0, 2, 5, 10 and 20, followed by RT-qPCR. It was observed that, by DAI 5, *Ptchd1-a* exhibited elevated expression relative to DAI 0. Furthermore, *Ptchd1-a* levels continued to increase until DAI 20, culminating in a 20-fold increase relative to DAI 0 at this terminal time point (Fig. 1B). We next sought to replicate the activity-dependent neuronal inductions of *Ptchd1* and *PTCHD1* by Kim et al.^13^ and Ross et al.^14^, respectively. Following 6 h of KCl-mediated depolarization of neurons at DAI 20, we observed a modest (albeit not statistically significant) 35% augmentation in *Ptchd1-a* expression (p = 0.095; student’s unpaired t-test, d.f. = 2) (Fig. 1C).

### Ptchd1 promoter sequence analysis

**Figure 2:**
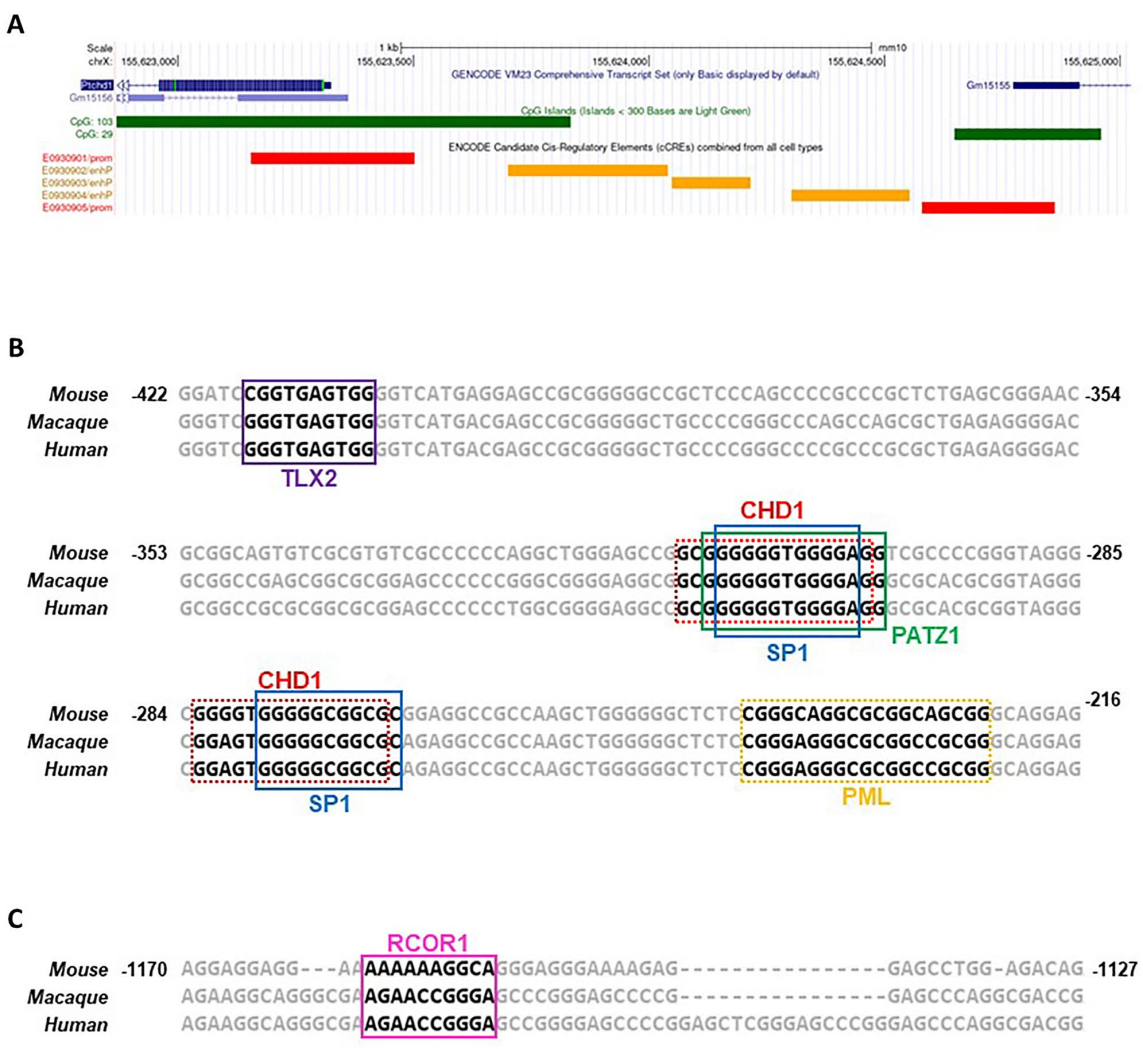
Proximal enhancer elements and evolutionarily conserved putative TFBSs within the *Ptchd1* promoter. (**A**) UCSC genome browser image of the mouse *Ptchd1* promoter (GRCm38) indicating annotations corresponding to the core promoter (red), CpG islands (green) and proximal enhancers (orange). (**B**) Evolutionarily conserved putative binding sites for the transcription factors TLX2, Sp1, CHD1, PATZ1 and PML within the *Ptchd1* promoter between − 422 and − 216 relative to the TSS. The sequence alignment for the mouse, macaque and human genomes is displayed, with the relative coordinates pertaining to the mouse sequence. Solid rectangles indicate the motifs for transcription factors predicted to bind on the forward strand, and dashed rectangles indicate those binding to the reverse strand. (**C**) Evolutionarily conserved putative binding site for the transcriptional corepressor RCOR1 within the *Ptchd1* promoter between − 1170 and − 1127 predicted to bind to a motif on the forward strand.

The *PTCHD1* promoter exhibits substantial sequence similarity between the human, rat, macaque and mouse genomes. This similarity includes an uninterrupted ECR in the mouse, rat and human genomes that encompasses over 1100 bp immediately upstream of the CDS (Fig. S2). This ECR exhibits 81% sequence identity between the human and mouse genomes (Fig. S3). Furthermore, at the nucleotide level in mice, the *Ptchd1* promoter harbours a large CpG island, as well as three annotated proximal enhancer elements: EM10E0930902, EM10E0930903 and EM10E0930904 (Fig. 2A). The CpG island is characterized by an approximately 350 bp region with an average GC content of over 80% (Fig. S4). *Ptchd1* appears to share an overlapping, bidirectional promoter with the annotated non-coding gene *Gm15155* (ENSMUSG00000055109) (Fig. 2A), which has a TSS that is less than 1.5 kbp upstream from *Ptchd1* CDS and exhibits 42% sequence conservation with the syntenic human long non-coding RNA gene *PTCHD1-AS* (ENSG00000233067) (Fig. S5). In contrast, 94% and 98% sequence identity exists at the nucleotide and protein levels, respectively, between the mouse and human orthologues of *PTCHD1*.

### Promoter basal and depolarization-mediated luciferase activity, and putative TFBSs

**Figure 3:**
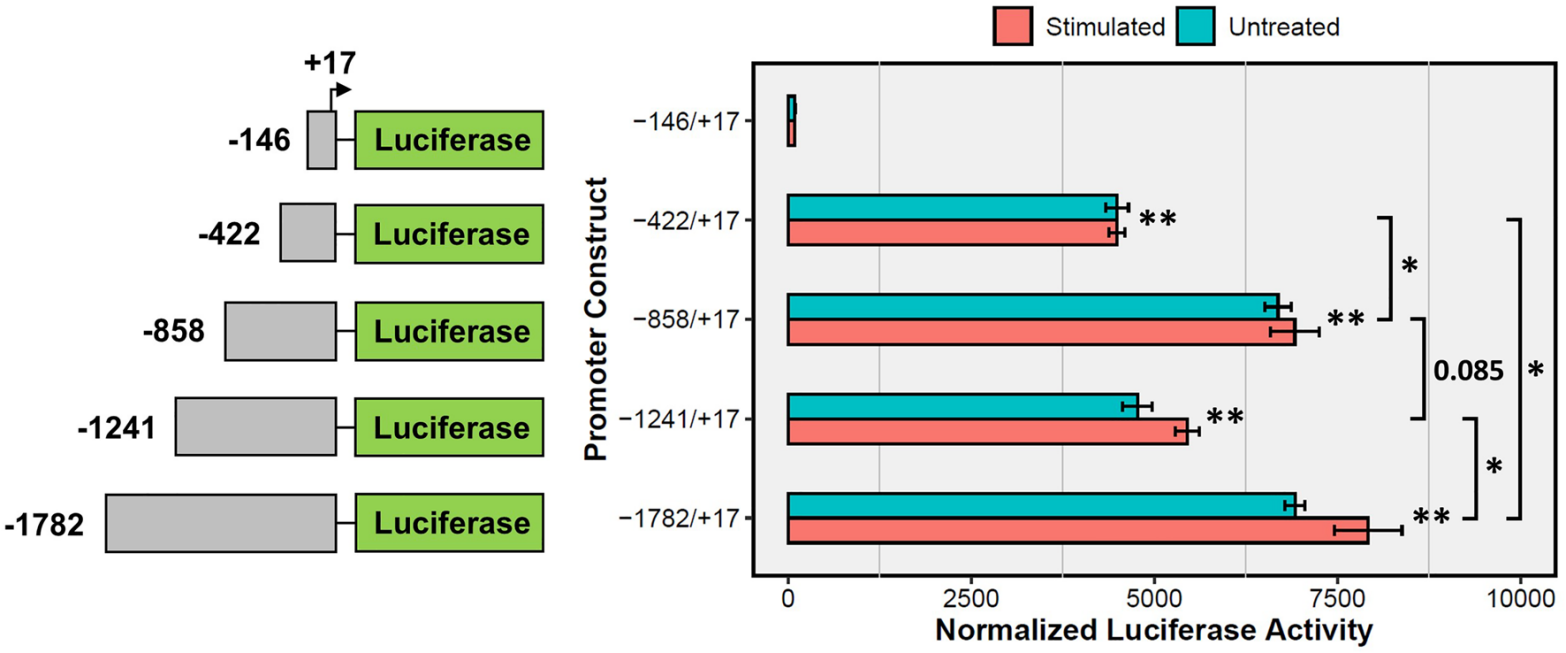
Luciferase activity of *Ptchd1* promoter 5′ truncations. Luciferase activity for P19 cell lines stably-expressing the reporter cassettes with 5′ truncations of the *Ptchd1* promoter ranging from -1782 to + 17 relative to the TSS at DAI 16. Data are expressed as the mean ± SEM for each group and were analyzed using a one-way ANOVA followed by a Tukey’s HSD test (**p < 0.001 relative to the − 146 to + 17 construct, *p < 0.05 between the indicated groups; n = 3 separate clones analyzed for each construct).

P19 cell lines stably-expressing reporter constructs with *Ptchd1* promoter 5’ truncations (Fig. 3) were differentiated until DAI 16, at which point luciferase activity was measured in both untreated and depolarized neurons. No differences were observed for luciferase activity as a consequence of depolarization for any of the promoter truncations evaluated in this study (Fig. 3). Furthermore, while essentially no luciferase activity was driven by the promoter construct spanning from − 146 to + 17 relative to the TSS, a 48-fold enhancement was observed when the preceding 276 bp were present (Fig. 3). This 276 bp region harbours putative binding-motifs for the transcription factors Specificity protein 1 (Sp1), T-cell leukemia homeobox protein 2 (TLX2), chromodomain helicase DNA-binding protein 1 (CHD1), POZ-, AT hook-, and zinc finger-containing protein 1 (PATZ1), as well as promyelocytic leukemia protein (PML), whose TFBSs within the promoter are all highly conserved between the mouse, macaque and human genomes (Fig. 2B). A further 49% increase in luciferase activity was evident when the upstream 436 bp were included (Fig. 3). Interestingly, inclusion of the region just upstream to this, encompassing − 1241 to − 859 bp, attenuated luciferase activity by 29% (p = 0.085; student’s unpaired t-test, d.f. = 2) (Fig. 3). This promoter segment harbours a conserved predicted binding-site for restrictive element 1-silencing transcription factor corepressor 1 (RCOR1) (Fig. 2C). Luciferase activity was fully restored (104% relative to the − 858/ + 17 construct) when the final 541 bp, spanning from − 1782 to − 1242, was incorporated into the construct (Fig. 3). This data was generated using a polyclonal approach to eliminate potential biases for monoclones, as discussed in Methods. Additional efforts using a monoclonal approach, however, gave results very similar to those presented in Fig. 3 (Fig. S6).

### Identification, analysis and validation of a putative downstream regulatory element

**Figure 4:**
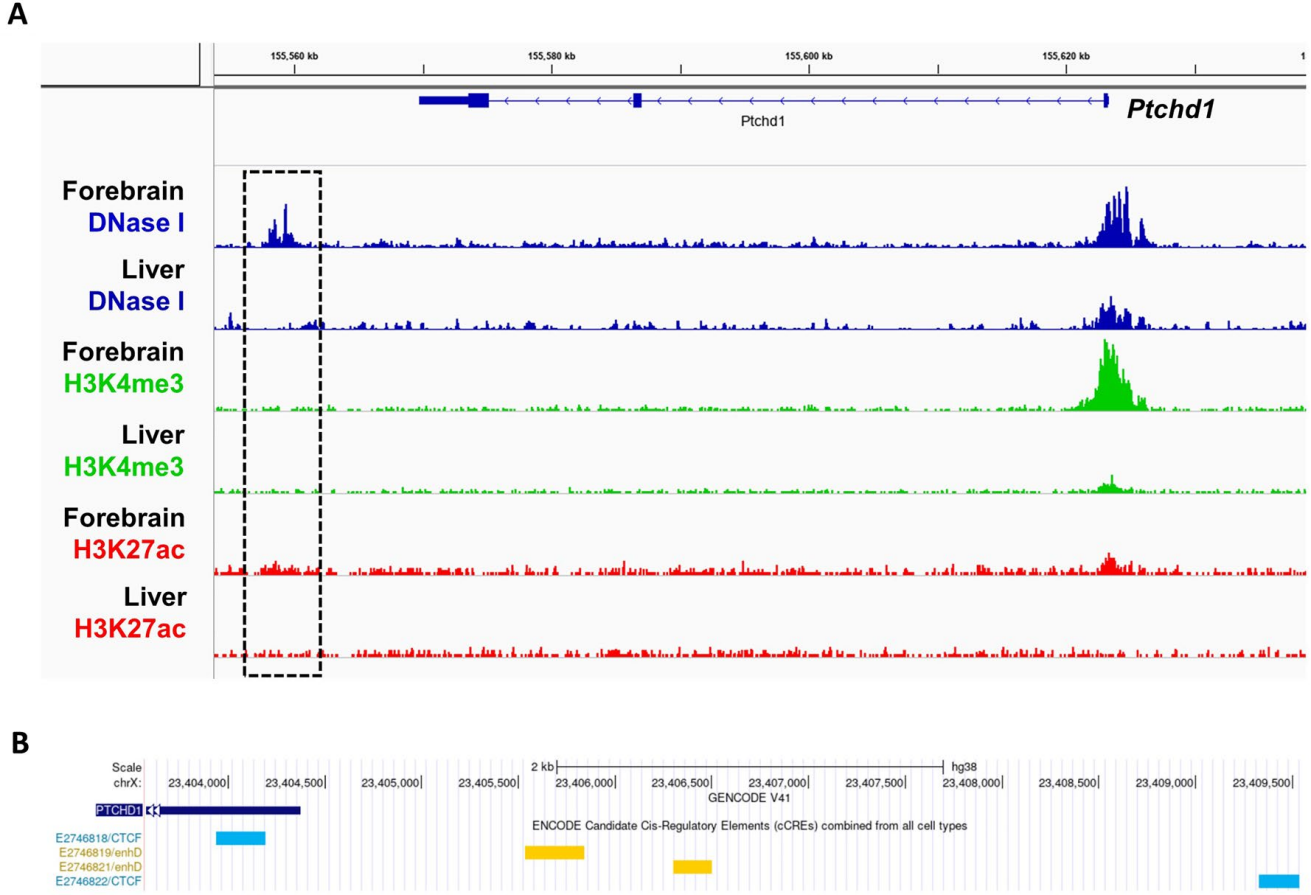
Open chromatin regions and active enhancers peripheral to *Ptchd1*. (**A**) Alignments from DNase-seq. (blue), H3K4me3 ChIP-seq. (green) and H3K27ac ChIP-seq. (red) experiments from the mouse P0 forebrain and liver. The dashed box corresponds to the putative downstream regulatory region. Call sets uploaded by Prof. Stamatoyannopoulos (University of Washington) were downloaded from the ENCODE portal^26^ (encodeproject.org/) with the following identifiers: ENCSR791AJY (forebrain; DNase-seq), ENCSR258YWW (forebrain; H3K4me3 ChIP-seq), ENCSR094TTT (forebrain; H3K27ac ChIP-seq), ENCSR216UMD (liver; DNase-seq), ENCSR653AVN (liver; H3K4me3 ChIP-seq) and ENCSR616TJM (liver; H3K27ac ChIP-seq). (**B**) UCSC genome browser image of region downstream of the human *PTCHD1* transcript (GRCh38) indicating annotations corresponding to CTCF-mediated silencers (light blue) and distal enhancers (yellow).

**Figure 5:**
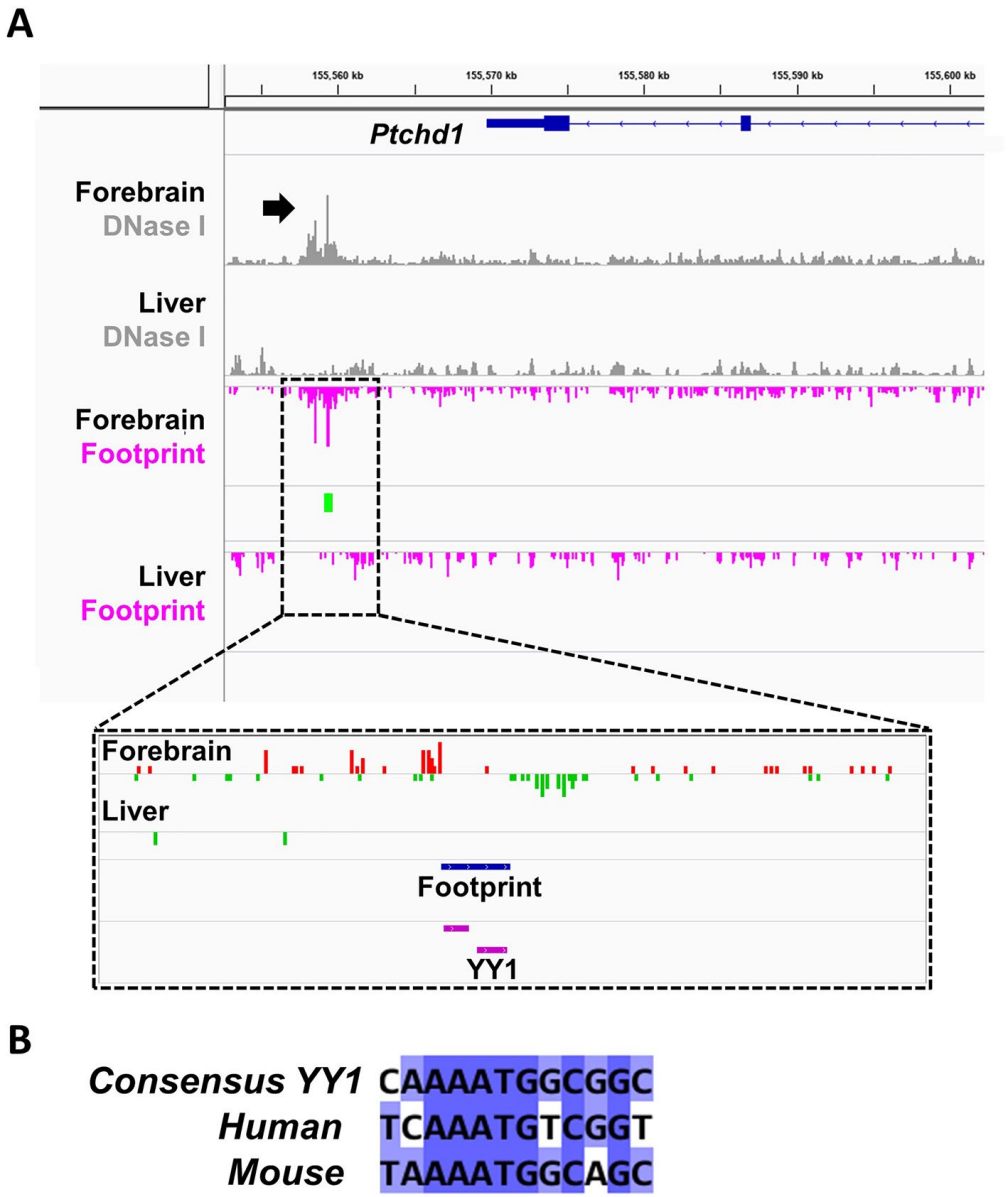
DNase footprint analyses for open chromatin regions peripheral to *Ptchd1*. (**A**) Alignments from DNase-seq. (gray) experiments from the mouse P0 forebrain and liver with the downstream open chromatin regions identified (black arrow) above the corresponding DNase footprint analyses (magenta) and specific DNase footprints (green). A higher magnification read alignment for the DNase footprint within the downstream putative regulatory region showing the characteristic accumulation of reads on opposite strands surrounding the protein-binding sequence (*inset*). (**B**) DNA alignment between the consensus binding site for YY1, the predicted YY1-binding site in the mouse DNase footprint, and the homologous genomic region in human.

DNase-seq read alignments from the mouse P0 forebrain and liver datasets revealed a mutual highly-accessible region of chromatin corresponding to the *Ptchd1* promoter in both tissues (Fig. 4A). DNase-seq signal peaks within the promoter were more pronounced in the forebrain relative to the liver, which overlaps with ChIP-seq. data of the epigenetic marker H3K4me3 (Fig. 4A), a marker of active promoters. Conversely, there was a unique region of highly-accessible DNA approximately 8 kbp in length located approximately 9.1 kbp downstream from the *Ptchd1*﻿ stop codon, that was present in the forebrain, but notably absent in the liver (Fig. 4A), as well as absent in the midbrain and hindbrain (Fig. S7), and in peripheral tissues (liver, heart, kidney, stomach and intestine; Fig. S8). This homologous downstream genomic region was concordantly accessible to DNase I in the human embryonic brain, but not in the liver (Fig. S9). In humans, the genomic sequence homologous to this downstream region contains two annotated distal enhancers, EH38E2746819 and EH38E2746821 (Fig. 4B), that exhibit 56% and 61% sequence identity, respectively, between human and mouse (Fig. S10). These two enhancers are also flanked by two annotated CCCTC-binding factor (CTCF) elements, EH38E2746818 and EH38E2746822, which display 71% and 63% sequence identity, respectively, between the human and mouse genomes (Fig. 4B). DNase footprint analyses in the mouse forebrain indicated that this 8 kbp downstream open chromatin region contained a 30 bp DNase footprint (chrX: 154,342,343–154,342,372; GRCm39), and subsequent motif discovery algorithms predicted that this DNase footprint possessed a putative binding site for the transcriptional activator-repressor Yin Yang 1 (YY1^30^) (chrX: 154,342,360–154,342,371; GRCm39) (Fig. 5A). This binding motif is partially conserved in humans (Fig. 5B), but notably contains an adenine instead of a cytosine residue at the second position of the core canonical YY1 consensus binding sequence^30^ (Fig. S11).

**Figure 6:**
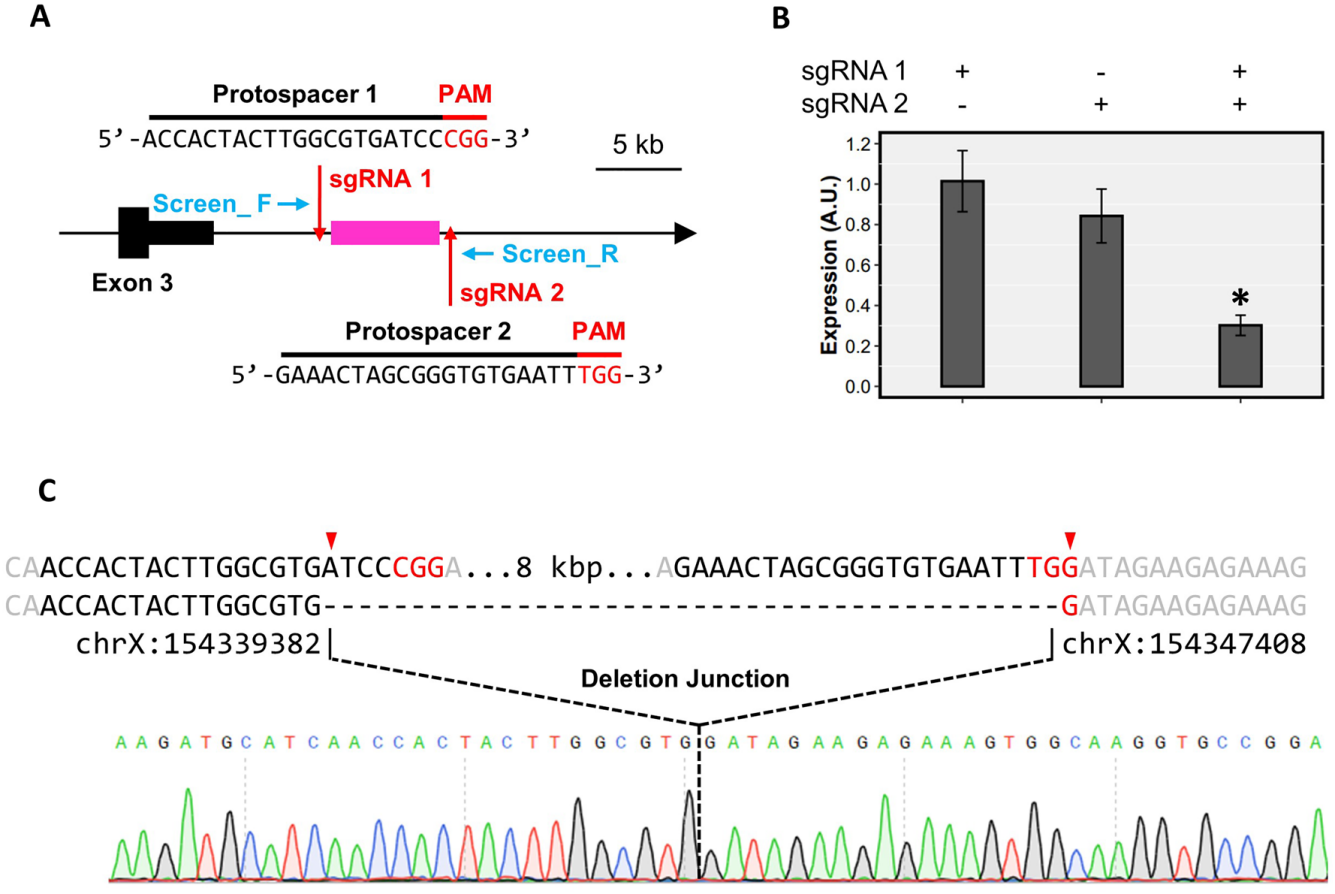
Contribution of putative downstream regulatory region on *Ptchd1* expression. (**A**) Schematic of *Ptchd1* depicting locations of the CRISPR-Cas9 protospacers (red arrows) and screening primers (blue arrows) used to delete the putative downstream regulatory region (magenta). (**B**) *Ptchd1-a* mRNA levels in P19 clones with deletion of the putative downstream regulatory region (both sgRNA 1 and sgRNA 2) and non-deletion controls (sgRNA 1- or 2-only) at DAI 16. Data are expressed as the mean ± SEM for each group and were analyzed using a one-way ANOVA followed by a Tukey’s HSD test (*p < 0.05 relative to both the sgRNA 1- or 2-only lines; n = 3 separate clones analyzed for each experimental condition). (**C**) Confirmation of deletion of the putative downstream regulatory element. The top strand contains the genomic DNA sequence immediately surrounding the deletion break points, including the protospacer (black), PAM (red) and flanking bases (gray), and the bottom strand displays the unique deletion junction of one of the clones above its electropherogram.

In order to ascertain the effects of this prospective downstream regulatory element on the neuronal expression of *Ptchd1-a*, we utilized CRISPR-Cas9 to delete this genomic region (Fig. 6A,C). To mitigate inter-clonal variability, non-deletion control lines were generated by transfection with only sgRNA 1 (control 1) or sgRNA 2 (control 2) and used for comparison with deletion clones. At DAI 16, deletion clones exhibited 70% and 64% reductions in *Ptchd1-a* transcription relative to control lines 1 and 2, respectively (Fig. 6B).

Taken together, these data have ascertained critical segments within the *Ptchd1* promoter, as well as identified conserved TFBSs within these regions that may be regulating its transcription. In addition, an enhancer-containing putative regulatory region downstream of *Ptchd1* was found, deletion of which significantly attenuated expression.

## Discussion

Perturbations in the expression of dosage-sensitive genes are strongly implicated in the etiology of ASD^31^, with de novo CNVs accounting for up to 8% of idiopathic ASD cases^32^. The disease-causing mechanisms of non-hemizygous CNVs are presumably related to altered gene dosage during critical periods of neurodevelopment that canonically rely on precise levels of gene expression^33^. Putative dosage-sensitive genes have been identified for several CNV-derived syndromic forms of ASD and ID. For example, Smith-Magenis Syndrome (OMIM: 182290) and Potocki-Lupski syndrome (OMIM: 610883) involve a reciprocal microdeletion and microduplication, respectively, at the locus 17p11.2 that affects the dosage-sensitive gene Retinoic acid-induced 1 (*RAI1*) (MIM: 607642)^34,35^. In addition, Phelan-McDermid Syndrome (OMIM: 606232) and 22q13 Duplication Syndrome involve a reciprocal microdeletion and microduplication, respectively, of the locus 22q13.3, which encodes the dosage-sensitive gene SH3 and multiple ankyrin repeat domains 3 (*SHANK3*) (MIM: 606230)^36–38^. Interestingly, a multitude of both inherited and de novo microdeletion CNVs affecting all or part of the *PTCHD1* CDS have been associated with ASD and ID^6^. In addition to CNVs, a selection of common variants that do not affect the *PTCHD1* CDS but are predicted to alter its expression have also been linked to the etiology of ASD^15^, suggesting that *PTCHD1* may be a dosage-sensitive gene that is important in neurodevelopment. The present study sought to explore the sequence-specific factors at the *Ptchd1* locus that affect its expression in a mouse neuronal model in vitro. Specifically, we characterized both basal and depolarization-induced *Ptchd1* promoter activity, as well as identified and validated a downstream putative regulatory region.

The model system for this study is the male mouse-derived pluripotent embryonal carcinoma cell line P19^16^. This cell line was selected because it was derived from a male mouse, and is therefore hemizygous for *Ptchd1*. This characteristic allows P19 cells to be particularly amenable to genomic modifications at this locus, as there is only one allele that needs to be modified. In addition, methods of differentiation of P19 cells and subsequent enrichment for neuronal cells have been well-established^20^. Despite these advantages, there are potential limitations regarding the clinical translatability of findings obtained from a mouse model. These limitations are partly mitigated due to the strong sequence homology between the human and mouse *Ptchd1* CDS, proximal promoter, and annotated downstream enhancer elements. Also, a pairwise comparison between human embryonic forebrain and liver DNase-seq. data reveals a homologous open chromatin region downstream of the *PTCHD1* 3’ UTR in the forebrain that is absent in the liver (Fig. S9). The concordance in DNA accessibility in these homologous regions between human and mouse supports the clinical relevance of the regulatory elements identified and analyzed in this study. *Ptchd1* expression increased exponentially with neuronal differentiation, attaining a 20-fold induction at DAI 20 (Fig. 1B). This result is consistent with the finding that *PTCHD1* demonstrates its highest levels of expression in the brain in comparison with peripheral tissues^6^, and is also concordant with its putative role as an ASD and ID susceptibility gene and a role in neurodevelopment. Surprisingly, a robust activity-mediated induction of *Ptchd1* was not observed in this study (Fig. 1C), as has been reported in other studies^13,14^. Similarly, we did not detect significant elevations in luciferase activity after depolarization in any of the promoter constructs that were examined (Fig. 3B). As we only investigated segments of the *Ptchd1* promoter that extended as far as − 1782 relative to the TSS, this outcome suggests that the depolarization-responsive cis-regulatory elements that modulate expression of *Ptchd1* may be located more distally to the TSS. This explanation is substantiated by the finding that most depolarization-dependent binding sites for the transcriptional coactivator cyclic AMP (cAMP) response element-binding protein (CREB)-binding protein (CBP), which binds exclusively to enhancers^39^, are located more than 2 kbp distal to TSSs^13^.

Candidate cis-regulatory elements with promoter-like signatures (cCRE-PLS) are characterized by both strong DNase and H3K4me3 signals, and are also located within 200 bp of an annotated TSS. Accordingly, a 347 bp cCRE-PLS is present at the *Ptchd1* locus between − 177 and + 170 relative to the TSS (chrX: 154,406,152–154,406,498; GRCm39) (Fig. 2A). In this vein, the comparatively miniscule level of luciferase activity in the smallest promoter construct, − 146 to + 17, may be attributable to the absence of upstream components of the core *Ptchd1* promoter in the 31 bp region between − 177 and − 147. More broadly, mutations within the core *Ptchd1* promoter may be particularly deleterious, as both the 27 bp duplication (chrX: 23,334,788–233,347,90; GRCh38) and GCC microsatellite (chrX: 23,334,788–23,334,820; GRCh38) reported by Torrico et al.^15^ are located at regions of the *PTCHD1* locus that are homologous to the annotated mouse cCRE-PLS.

The promoter region enhancers that are most essential for transcription of *Ptchd1* appear to be located between − 422 and − 147 upstream of the TSS (Fig. 3). To corroborate this assertion, we sought to identify putative TFBSs within this 276 bp region that show evolutionary conservation between the mouse, macaque and human genomes. Conserved predicted binding motifs for the transcription factors TLX2, Sp1, CHD1, PATZ1 and PML were observed in this region (Fig. 2B). CHD1 has been found to bind directly to H3K4me3^40^ and has been implicated in the assembly of active chromatin in vitro^41^, supporting the notion that binding at this location may increase the accessibility of the preinitiation complex (PIC) to elements within the *Ptchd1* promoter. In addition to CHD1, this region of the promoter contains two conserved predicted binding sites for the ubiquitously-expressed transcription factor Sp1, which facilitates formation of the PIC by binding to the TATA box-binding protein (TBP)^42^. Furthermore, the transcriptional activator TLX2 is reportedly expressed in tissues derived from neural crest cells^43^ and exhibits upregulation in C1300 and SH-SY5Y neuroblastoma cell lines following stimulation with RA^43^, suggesting that TLX2 could directly facilitate *Ptchd1* expression during differentiation. Conversely, PATZ1 contains a POZ domain, which is associated with mediating transcriptional repression^44^, making it unlikely that it would facilitate the robust augmentation of luciferase activity conferred by this 276 bp sequence. Interestingly, PML is a highly-pleiotropic protein that has been implicated in both transcriptional activation and repression^45^, and binding within this 276 bp segment could promote the expression of *Ptchd1* under appropriate circumstances. Reciprocally, the slight decrease in luciferase activity that was yielded by the − 1241 to − 859 region could be explained by a conserved predicted binding motif for the transcriptional corepressor RCOR1^46^ (Fig. 2C). Moreover, bidirectional promoter interference has been documented previously^47^, and therefore the region encompassing upstream of − 859 could contain proximal elements or inhibitory chromatin modifications that pertain to the *Gm15155* promoter which may also suppress *Ptchd1* transcription. Looking at ASD-associated SNVs from available whole genome sequence from the MSSNG dataset (research.mss.ng), we identified 56 rare SNVs in affected males within these conserved TFBSs, including one variant (MSSNG ID # REACH000589) within the RCOR1 binding motif (Table S5), which could potentially impact *PTCHD1* expression and thus be etiologically relevant. However, these variants include 18 within multiplex families, but we observed no excess of segregation versus non-segregation of these variants with affected status within these families. Additional studies would be needed to determine whether any of these variants impact *PTCHD1* expression, thus potentially contributing to the ASD etiology.

In this study, mouse P0 forebrain and liver tissues were chosen for pairwise comparison because they exhibit opposing levels of *Ptchd1* transcription^6^. Correspondingly, DNase-seq. data reveals a disparate level of DNA accessibility downstream of *Ptchd1*, with an enhancer-containing region of open chromatin being apparent in the forebrain but conspicuously absent in the liver (Figs. 4 and 5). In addition, DNase-seq. data from the mouse P0 midbrain and hindbrain reveal that this open chromatin region exists exclusively in the forebrain (Fig. S7). This result suggests that Ptchd1 may be involved in the early development of forebrain-derived components of the central nervous system, including the thalamus and cerebral cortex.

The enhancer EH38E2746819 from the region just downstream of human *PTCHD1* contains a large inverted repeat sequence that has the potential to form unusual secondary structures, such as a stable DNA cruciform (Fig. S12). Such DNA cruciform structures have been shown to occur in vivo, and are important for a range of biological functions, including transcriptional regulation and nucleosome structure^48^. Moreover, we have identified a DNase footprint within the mouse *Ptchd1* downstream region in the mouse forebrain that contains a putative binding motif for the transcriptional activator-repressor YY1 (Fig. 5A). Functionally, YY1 dimerization has been demonstrated to catalyze structural interactions between promoters and distal enhancers through DNA looping^49^. However, we unexpectedly did not identify a corresponding DNase footprint with a YY1 binding motif in the *Ptchd1* promoter that could potentially mediate this interaction. This absence of an inferred YY1 binding site in the *Ptchd1* promoter could be due to the fact that YY1 binding may be either extremely transient or displaced during formation of the preinitiation complex, and therefore unable to be captured by the DNase-seq. assay. Despite this, there is evidence of a possible YY1 binding motif located in the *Ptchd1* promoter that is conserved in both the mouse (chrX: 154,406,481–154,406,486; GRCm39) and human (chrX: 23,334,714–23,334,719; GRCh38) genomes (Fig. S2) which could potentially facilitate structural interaction with YY1 bound within the downstream open chromatin region. Conversely, YY1 has been demonstrated to heterodimerize with the transcription factor Sp1^50^, which contains two conserved TFBSs within the *PTCHD1* promoter (Fig. 2B), thereby providing an alternative mechanism for interaction of the downstream topologically-active domain (TAD) with the promoter. In addition, CTCF has been implicated in demarcating the boundaries of TADs via interactions with the cohesin protein complex^51^. Collectively, these data suggest a mechanism whereby enhancer elements within an extruded TAD downstream of *PTCHD1/Ptchd1*, when accessible and in proximity to the *PTCHD1*/*Ptchd1* promoter through YY1-mediated DNA looping, may contribute to increased *PTCHD1/Ptchd1* expression. Removal of these conserved enhancers and putative YY1 binding motif through deletion of the entire downstream open chromatin region significantly attenuated *Ptchd1* expression in our neuronal model (Fig. 6B), validating its predicted regulatory function. Clinically, numerous rare SNVs associated with ASD and ID are located within the downstream enhancer and CTCF elements (Table S5) (PMID 28263302), suggesting that these regions are relatively intolerant to mutations.

While evidence has implied that *PTCHD1* may be a dosage-sensitive gene whose reduced expression correlates with ASD and ID^15^, there is a paucity of research into mechanisms that govern its expression. This study provides a higher-resolution examination of the *Ptchd1* promoter, specifically elucidating a core segment containing proximal enhancers that are critical for its expression. Furthermore, we have also identified conserved putative TFBSs within the *Ptchd1* promoter that may mediate its transcription. We have also identified and validated a regulatory region situated downstream of *Ptchd1* that appears to contain enhancers, as well as proposing a mechanism whereby these distal enhancers may structurally interact with the core promoter. Collectively, these data will help clarify the relationship between the genomic regions surrounding *PTCHD1* and their effects on its expression in neurons, serving to provide critical context for structural variations in this region that may impact neurodevelopment.

### Supplementary Information

Supplementary Information.
