# Supplementary material for: Neuronal transcription of autism gene PTCHD1 is regulated by a conserved downstream enhancer sequence
Source: Sci Rep. 2023 Nov 21;13:20391. doi: 10.1038/s41598-023-46673-0 (PMC10663455; doi:10.1038/s41598-023-46673-0)

Supplementary Information

Table S1. Primers used to generate the modified pBV-Luc::hPGK-PuroR vector, as well as the *Ptchd1* promoter truncations. Restriction enzyme recognition sequences are underlined.

Primer	Sequence
NotI_hPGK_F	5'-TAGCTAGCGGCCGCTTGCGCCTTTTCCAAGGCAGC-3'
NotI_PuroR_R	5'-TAGCTAGCGGCCGCTCAGGCACCGGGCTTGCGGG-3'
BamHI_-146_F	5'-TACGGGATCCTCCAGGAGAGCTCAGGGTCTTG-3'
BamHI_-422_F	5'-TACGGGATCCGGTGAGTGGGGTCATG-3'
HindIII_+17_R	5'-TACGAAGCTTCCTAGAGCAGAGCGGGCG-3'
KpnI_-858_F	5'-TACGGGTACCCATGCTCGAGCGTCTCGGTTC-3'
KpnI_-1241_F	5'-TACGGGATCCGGGGCCCGCACCAGCTC-3'
KpnI_-1782_F	5'-TACGGGATCCCCAGCGGTGTGGAGTGGG-3'
BamHI_-417_R	5'-TACGGGATCCCCGAAGCCAACGAC-3'

Table S2. Primers and cDNA dilutions used for qPCR.

Primer	Sequence	Efficiency	cDNA Dilution	Reference
Ptchd1-a_Exon1-2_F	5'-CCTGATCTTAAAGTTGCATACTGC-3'	1.89	1:2	[6]
Ptchd1-a_Exon2_R	5'-GTGGCCCGAGCATTCTTTAG-3'			
β-Actin_F	5'-AGGCCAACCGTGAAAAGATG-3'	2.02	1:500	[25]
β-Actin_F	5'-CACAGCCTGGATGGCTACGT-3'			
Gapdh_F	5'-TGTGTCCGTCGTGGATCTGA-3'	2.05	1:500	[25]
Gapdh_R	5'-CCTGCTTCACCACCTTCTTGA-3'			

Table S3. Primers used to delete the downstream open chromatin region in P19 cells. Protospacer sequences are underlined.

Primer	Sequence
Protospacer_1_F	5'-CACCGACCACTACTTGGCGTGATCC-3'
Protospacer_1_R	5'-AAACGGATCACGCCAAGTAGTGGT-3'
Protospacer_2_F	5'-CACCGGAAACTAGCGGGTGTGAATT-3'
Protospacer_2_R	5'-AAACAATTCACACCCGCTAGTTTC-3'
Deletion_Screen_F	5'-GGTAGCCAAACATTTTGCATTGG-3'
Deletion_Screen_R	5'-GTATGTGTGCTCCTCCAACAC-3'

Table S4. Genomic coordinates of UCSC-annotated human *PTCHD1* and inferred mouse *Ptchd1* downstream distal enhancer elements.

Identifier	Element	Annotated Species	Coordinates (GRCh38)	Inferred Coordinates (GRCm39)
EH38E2746819	Enhancer	Human	23,405,534-23,405,842	154,341,292-154,341,585
EH38E2746821	Enhancer	Human	23,406,301-23,406,501	154,340,688-154,340,914
EH38E2746818	CTCF	Human	23,403,939-23,404,195	154,343,364-154,343,633
EH38E2746822	CTCF	Human	23,409,330-23,409,538	154,337,302-154,337,507

Figure S1. Plasmid diagram for the generation of stably-transfected cell lines expressing reporter vectors with 5' truncated *Ptchd1* promoter constructs.

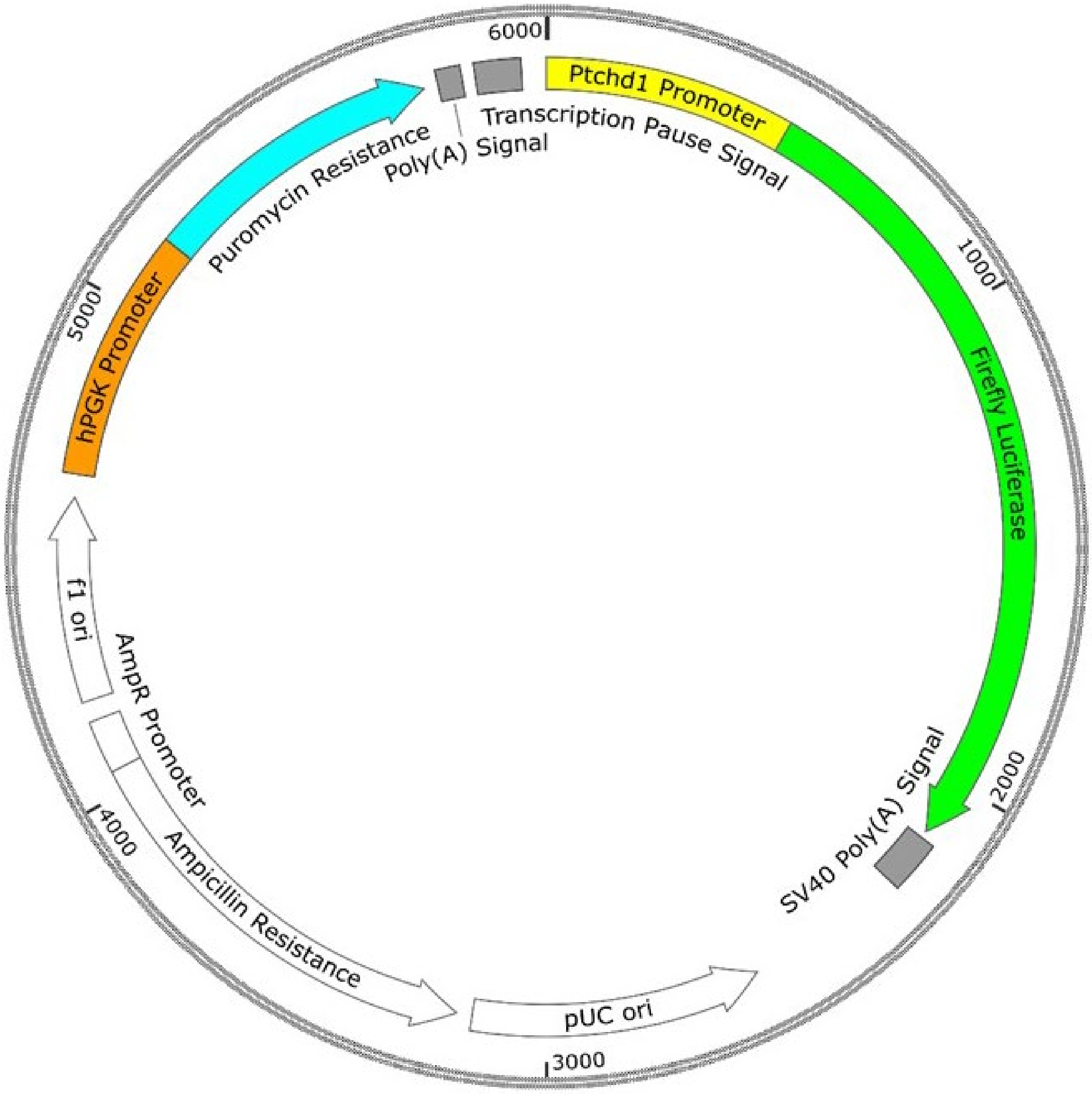

Figure S2. Conservation of human *PTCHD1* promoter with mouse, rat and macaque.
ECR browser image showing ECRs (pink horizontal bar) between the human *PTCHD1* promoter and the corresponding orthologous promoters in rat, mouse and macaque. The conserved core canonical YY1 binding motif within the promoter is identified for the human, rat and mouse genomes (*inset*).

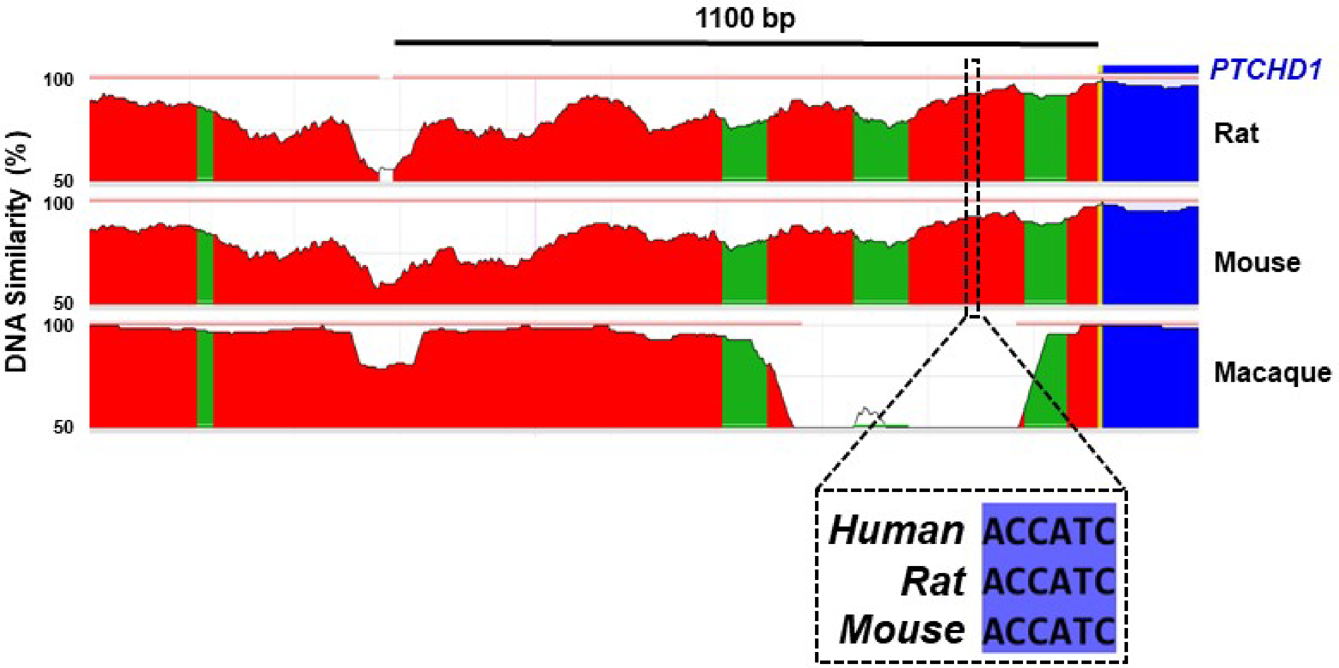

Figure S3. Sequence alignment of the ECR of the human and mouse orthologs of the *PTCHD1* promoter. The UCSC-annotated 5' UTR is highlighted in yellow.

```
#=======================================
#
# Aligned_sequences: 2
# 1: Human
# 2: Mouse
# Matrix: EDNAFULL
# Gap_penalty: 10.0
# Extend_penalty: 0.5
#
# Length: 1173
# Identity:     950/1173 (81.0%)
# Similarity:   950/1173 (81.0%)
# Gaps:          72/1173 ( 6.1%)
# Score: 3905.0
#
#
#=======================================


Human              1 GGAGCCCGGGAGCCCAGGCGACGGACAGTTCCATCGACTGACC----GTC     46
                             |||| ||.||.|||.|||||||||||.||||||||    |||
Mouse              1 --------GGAG-CCTGGAGACAGACAGTTCCATGGACTGACCGAGTGTC     41

Human             47 ATTATCAGCACTTTTATTACTAAAGGACTGGGTGG-GAGGTTGG----GG     91
                     |||||||||||||||.|||.|...||||.|||||| |||..|||    ||
Mouse             42 ATTATCAGCACTTTTCTTATTGGGGGACAGGGTGGAGAGAGTGGTGAAGG     91

Human             92 GGTGTGCAACAGAGCTGGCCAGCCCCAATCCACTCTGGAGCGACCCTAAA    141
                     |.| ||||.||   |||.|||| ..|||||||||||||||||||||||||
Mouse             92 GAT-TGCACCA---CTGCCCAG-TTCAATCCACTCTGGAGCGACCCTAAA    136

Human            142 TTAGAGCCAC---AGAGAAAGATTCTCCACGGATACCTTTTGAGTGGACG    188
                     .|.|.|  ||   .|||.||||||.|||..|.|.||.|||.|.||||||.
Mouse            137 ATTGGG--ACTTGGGAGGAAGATTGTCCTAGAACACATTTGGGGTGGACA    184

Human            189 TGCTCCAGA--CACACACCCGGACCCC-GTGGTCCCGCCGAAGCTGCAGT    235
                     ..|||||.|  ||||||   ||.|||| |||||.||.|.|.||||||||
Mouse            185 GCCTCCAAATGCACACA---GGTCCCCTGTGGTGCCCCTGGAGCTGCAGT    231

Human            236 GTTGGGGGTATTTTAAACGGTTCTTATTTGGAAGGAGGCGTGATTTCCAC    285
                     |||||||..||.|||||.||.|||||.||||.|||||||||||||||||.
Mouse            232 GTTGGGGAGATCTTAAAAGGCTCTTACTTGGGAGGAGGCGTGATTTCCAT    281

Human            286 GTTCGAGCGTCTTCGCTCCCCACCCCCTCTCGGCACCAGCCAGTGCGCGC    335
                     |.||||||||||..|.|||||||||||||||||.|..|||||||||||||
Mouse            282 GCTCGAGCGTCTCGGTTCCCCACCCCCTCTCGGTAATAGCCAGTGCGCGC    331

Human            336 CACCACCTGAGCCTGGCGAGAGCCGGGACGCACGCAGTCTTCAGCCTGCT    385
                     |||||||||||||||||.|||||||||.|||||||||||||||||||||.
Mouse            332 CACCACCTGAGCCTGGCAAGAGCCGGGGCGCACGCAGTCTTCAGCCTGCG    381

Human            386 CCTTTAAGCCTCTGGAGAGACTCGGCTCTGAGCGGGTCCGCCCGACACCC    435
                     .||||..||.||||.|||||||.|||||..||||||||.|||||||||||
Mouse            382 TCTTTCCGCGTCTGAAGAGACTTGGCTCAAAGCGGGTCTGCCCGACACCC    431

Human            436 GCCCCCTCCCCCCACTCCTGAGAACTCTT-TACT---TTTTGTTTGGAAA    481
                     .|.||||.|||||...|..|||||||||| ||||   |||||||||.|||
Mouse            432 CCACCCTACCCCCTAACGGGAGAACTCTTATACTTTATTTTGTTTGAAAA    481

Human            482 ATCGGGGCCATTTCTTTATTTATCTCTGGGTCTTCCCGGAGGAGGGCA-G    530
                     |||.|||.|||.||||||||||||||||.||||.|||.|||||||.|| |
Mouse            482 ATCCGGGACATCTCTTTATTTATCTCTGAGTCTCCCCAGAGGAGGCCAGG    531
```

```
Human          531  AGAAGGGGA-------GAGCGAGCTTGGCGGGCCGGACCGGGGGA-GGGG   572
                    .||||||||       ||||..|||||||.||||...|.|.|||| ||||
Mouse          532  GGAAGGGGAAGGGGGTGAGCCCGCTTGGCCGGCCAAGCTGAGGGAGGGGG   581

Human          573  AGCGAAGGGAGGGGAGGGGACTGGGTGTAGCCTGGAATTCTCCGTCCTCG   622
                    ||...|..|||.||||||||||||||||||||||||||||||.|||||||
Mouse          582  AGTACACTGAGAGGAGGGGACTGGGTGTAGCCTGGAATTCTCTGTCCTCG   631

Human          623  TGTTCCTGGGCGGGCGCGCAGAGTCTCCTGGCGATAGCG------CCGTC   666
                    |||||| .|||.||||||||||||||.||||||.|||||      ||..|
Mouse          632  TGTTCC-AGGCTGGCGCGCAGAGTCTTCTGGCGCTAGCGCCACCACCACC   680

Human          667  GCCGCTCCCCGCGCCACCCCGTCGTTGGCCTTGGGGGTCGGGTGAGTGGG   716
                    .||||||.||||||||.||||||||||||.|.||||.||.||||||||||
Mouse          681  ACCGCTCTCCGCGCCATCCCGTCGTTGGCTTCGGGGATCCGGTGAGTGGG   730

Human          717  GTCATGACGAGCCGCGGGGGCTGCCCCGGGCCCCGCCCGCGCTGAGAGGG   766
                    |||||||.|||||||||||||.||.||..|||||||||||.|||||.|||
Mouse          731  GTCATGAGGAGCCGCGGGGGCCGCTCCCAGCCCCGCCCGCTCTGAGCGGG   780

Human          767  GACGCGGCCGCGCGGCGCGGAGCCCCCCTGGCGGGGAGGCCGCGGGGGGT   816
                    .|||||||.|.|..|||.|..|||||||.|||.|||||.|.|||||||||
Mouse          781  AACGCGGCAGTGTCGCGTGTCGCCCCCCAGGCTGGGAGCCGGCGGGGGGT   830

Human          817  GGGGAGGGCGCACGCGGTAGGGCGGAGTGGGGGCGGCGCAGAGGCCGCCA   866
                    |||||||.|||.|..||||||||||.|||||||||||||.||||||||||
Mouse          831  GGGGAGGTCGCCCCGGGTAGGGCGGGGTGGGGGCGGCGCGGAGGCCGCCA   880

Human          867  AGCTGGGGGGCTCTCCGGGAGGGCGCGGCCGCGGGCAGGAGGGCGTGTCT   916
                    |||||||||||||||||||..||||||||.||||||||||||||||||||
Mouse          881  AGCTGGGGGGCTCTCCGGGCAGGCGCGGCAGCGGGCAGGAGGGCGTGTCT   930

Human          917  CCGGCTCGAGGGGACCCTCCCGAGGCGCGGCGCGGGACTCCCCGATGGTG   966
                    ||.||..||||||.||||||||||||||||||||||||||||||||||||
Mouse          931  CCAGCCGGAGGGGTCCCTCCCGAGGCGCGGCGCGGGACTCCCCGATGGTG   980

Human          967  GTGCCGCGGCTCCGGGAGAGCTCAGGGTCT------CGCCGCCGCCGCCG  1010
                    |||||||||||||.||||||||||||||||      ||||||||||||||
Mouse          981  GTGCCGCGGCTCCAGGAGAGCTCAGGGTCTTGCCTCCGCCGCCGCCGCCG  1030

Human         1011  TCGCCGCCGCC---GCGGGCG---------CCGCTGCCGCCGCCGCCGCC  1048
                    |||||||||||   |||||||         ||.|..||.||.||||||||
Mouse         1031  TCGCCGCCGCCGCTGCGGGCGCCACCACCACCACCACCACCACCGCCGCC  1080

Human         1049  GCCGCCGCCGCCGCCGCCCGAACCCGCGCCGAGCGTGCGCCTCGCCCTCC  1098
                    |||||||||||||.|||||||.||||||||||||||||||||||||||||
Mouse         1081  GCCGCCGCCGCCGTCGCCCGAGCCCGCGCCGAGCGTGCGCCTCGCCCTCC  1130

Human         1099  TCCCGCGCCCGCTCTGCTCTAGG    1121
                    |||||||||||||||||||||||
Mouse         1131  TCCCGCGCCCGCTCTGCTCTAGG    1153
```

Figure S4. GC content of segments of the *Ptchd1* promoter relative to the TSS.

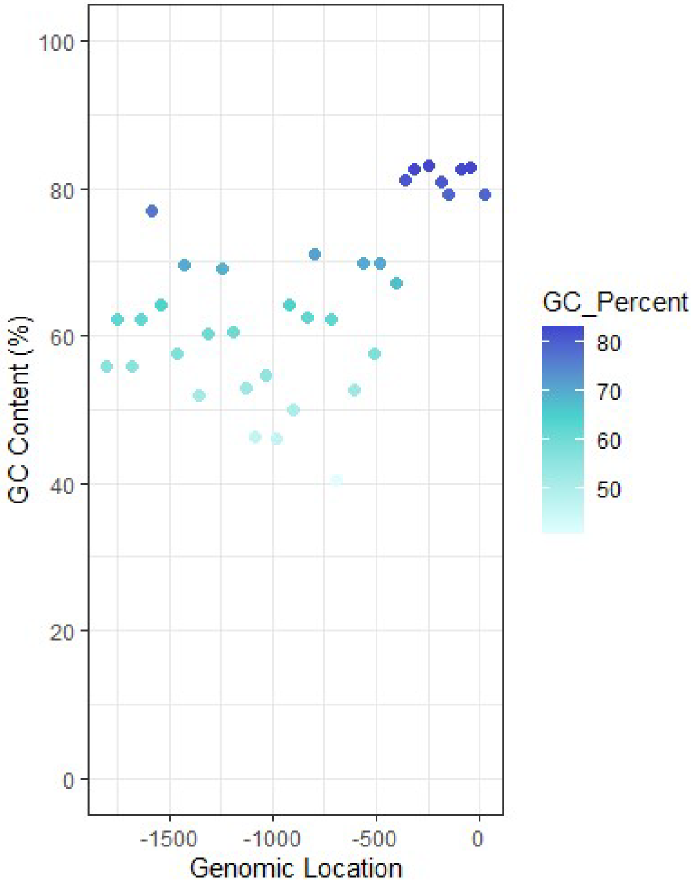

Figure S5. Sequence alignment of *PTCHD1-AS* and *Gm15155*.

```
#=======================================
#
# Aligned_sequences: 2
# 1: ENST00000669979.1 (PTCHD1-AS)
# 2: ENSMUST00000112542.2 (Gm15155)
# Matrix: EDNAFULL
# Gap_penalty: 10.0
# Extend_penalty: 0.5
#
# Length: 1430
# Identity:     603/1430 (42.2%)
# Similarity:   603/1430 (42.2%)
# Gaps:         645/1430 (45.1%)
# Score: 957.5
#
#
#=======================================
```

```
ENST000006699      1 TTGTTGGTGAATGTGATGCCTG--GTAG--AAGAGGCTCCTCAAGTCCCT     46
                     ||||..|||  |||| .|||.|  .|||  |..||||      ||.|.||
ENSMUST000001      1 TTGTGCGTG--TGTG-CGCCAGCCATAGCTAGCAGGC------AGCCACT     41

ENST000006699     47 G-------CCTTCTGTCATTTATCCATTTCCTGTAAGCCTGGTGAGAGAA     89
                     |       ||.||          .||.||||      |||.|.|||
ENSMUST000001     42 GTGGTTCCCCCTC----------GCACTTCC------CCTCGGGAG----     71

ENST000006699     90 GAAGAAGAGATGAGTCTCTGCAACGGTCCTCTCATTTATTGATTGCTGTA    139
                                                      ||||...||
ENSMUST000001     72 ---------------------------------ATTTCCCGA--------     80

ENST000006699    140 CTAAGTATTTGAA-ACTGACACTAGCAGCAGGGATATTCAGCCCAGGCCT    188
                               ||| .|.|||.||.||          |||      |||||
ENSMUST000001     81 ----------GAAGGCGGACGCTGGC----------TTC------GGCCT    104

ENST000006699    189 GGGAGCATGACCGAAGTCGGGCAGAAACTGCCATTCCTAACAGTGGCCAC    238
                     |||| || ||||...|.| |||.|.|||.||||   |..||||||..|
ENSMUST000001    105 GGGA-CA-GACCTGCGGC-GGCGGGAACGGCCA---CGCACAGTGATC--    146

ENST000006699    239 ACGAGGGCGGCTCTAAGAAGATGTGCCAGCTGGTAGTGAGCAGCCACAGC    288
                                 ||||||....|||            |||       ||.
ENSMUST000001    147 ------------CTAAGATCGCGTG------------GAG-------AGT    165

ENST000006699    289 CTTCCGGCTGTCCTCTGACCAGAGTCCTGTAGGAACAACGGC-CAGTATG    337
                     ||||.||        .|||.|.||..||||        |.|| .||| |.
ENSMUST000001    166 CTTCTGG--------GGACAATAGGACTGT--------CTGCTGAGT-TC    198

ENST000006699    338 GAGGACCCACGGCAGACTGGCCAACTGGCCAGAGGGA--AGGCAGGATGC    385
                     |.|  ||||.||     ||   |.||||  ||..|||  .|.|||||
ENSMUST000001    199 GTG--CCCAAGG-----TG---ACCTGG--AGCTGGAGCTGACAGGA---    233

ENST000006699    386 CTCCCTAAGGAGGTGGCCTGGCCTTTGAATAAAGATTTGA----AGGAAA    431
                           |||||.|||        ||||      |.|||||    ||.|
ENSMUST000001    234 ------AAGGAAGTG--------TTTG------GTTTTGATTCCAGAA--    261

ENST000006699    432 TGGGAGCATAAACCAGGCAG-GTCACTTGGGGAAGTTTTCCAAGAGAAAG    480
                            ||||||||..||| |||            .|.||.|.|| ||.
ENSMUST000001    262 -------ATAAACCAACCAGCGTC------------CTCCCTAAAG-AAT    291

ENST000006699    481 CCAACCC------CAGGTATAAATG---CTCCCAGAGCAGAGCGTGTCAG    521
                     |.||.||      || .|.||.|||   || .|||||.||||..||...|
ENSMUST000001    292 CAAAACCTTCTGACA-TTCTAGATGTGACT-ACAGAGAAGAGGATGAGTG    339
```

```
ENST000006699     522 GTGTGCTCTATAAACACGAAGGAGGCCAGTGTGGCCGGAACAGAAGGA--     569
                      ||.|||     |||||  ||              |..|||.|.||.||  
ENSMUST000001     340 GTTTGC-----AAACA--AA--------------CAAGAAAATAATGATC     368

ENST000006699     570 GGGAGGAAGGGGGA-----GAGTAGCAGGAGGTACAGGCAGAAAGA---T     611
                      .|||  ||.|.|||     |||| .||.||   ||||  .||||||   |
ENSMUST000001     369 TGGA--AATGTGGAGCTATGAGT-CCAAGA---ACAG--TGAAAGAGACT     410

ENST000006699     612 AACAGACCTTAAGGCCATTATAA---ATAATTT------GGCTCTTT---     649
                      .|||.|.| .|||...||.|.||   .||.|||      ||.||..|   
ENSMUST000001     411 CACATATC-AAAGAGAATGAGAAGCTGTATTTTTCAAGAGGATCACTAAA     459

ENST000006699     650 -----CTCTGAGTGAGATGGGATGCTGATAAAAGCTTTTGAGCAAAGAAG     694
                           ||||||||....|||.| ||.||.||||          |||||| 
ENSMUST000001     460 TACAGCTCTGAGTTTCCTGGTA-GCAGAAAAAA----------AAAGAA-     497

ENST000006699     695 TGACACGATGTGATTGTAAAACGTCTTAAAATCGCTACAATATTATATTT     744
                                      .|||||                 ||.|.|.|.||
ENSMUST000001     498 ----------------CAAAAC-----------------ATGTAACAGTT     514

ENST000006699     745 AGAATAGACTGAAGGGGG---GTAAGAACAAAAGCAGGGAGGCCA---AT     788
                        ||||.||   |.||||   ||.||    |.|||..||||.|||   .|
ENSMUST000001     515 --AATAAAC---ATGGGGCCTGTCAG----ATAGCCTGGAGTCCATCTCT     555

ENST000006699     789 GAGGAG-GCT-ATT-ACCATAATCCAGGTGGGAGAT--AGTAGTGGTATG     833
                      .|.||| ||| ||| |||           .|.||||  ||||.|..| |.
ENSMUST000001     556 CAAGAGAGCTGATTCACC-----------TGAAGATGAAGTACTTCT-TC     593

ENST000006699     834 AACAAGGGTGATAAATTCCATCCCCT------------------------     859
                      ..|     |.|.|||.||| ||||||                        
ENSMUST000001     594 TTC-----TCAGAAACTCC-TCCCCTGAGGTTTCTTTGTGTACCCTTAGC     637

ENST000006699     860 -------GCATTCAACACTGTAGA--AA---TGTGATTAAAACCA-----     892
                             |.|.|| ||.|||||||  ||   .||| ||.||..||     
ENSMUST000001     638 TGTCTTGGAACTC-ACTCTGTAGACGAAACTGGTG-TTGAACTCAGAGAT     685

ENST000006699     893 -------------------------TGAGATTAGA-TGT---------TA     907
                                               ||||||||.| |||         ||
ENSMUST000001     686 ATGCTTGCCTTTGCCTCCACAGTGCTGAGATTAAAGTGTGTGTGTGTCTA     735

ENST000006699     908 TCACC-------------------------------AAGCAAGTGAGCAA     926
                       ||||                               ||| ||||.|..||
ENSMUST000001     736 -CACCCCTCTCAGAAAAACGAAAACAACAAAAAAAAAAG-AAGTTAAAAA     783

ENST000006699     927 AGATGGAGAGAAGAGAACAGAAAAGGTGCAGACTGAGCCTTGGGGCATGC     976
                      |.|...|.||||.|.||..|||||||                        
ENSMUST000001     784 AAACTAAAAGAAAAAAATGGAAAAGG------------------------     809

ENST000006699     977 CATCATTAAGAGATATGGGAGATGAGGAGAAACCAGCAAGGATTTGAGCA    1026
                         ||.|||||.|.||      |||    ||||        ||      |
ENSMUST000001     810 ---CAATAAGACAAAT------TGA----AAAC--------AT------A     832

ENST000006699    1027 ACAGTGGCCCACCCTCCCCAAGACAAGC--CACCACCAACCGGAAT----    1070
                      |    |||.|||             |||  ||.||.||||   |||    
ENSMUST000001     833 A----GGCACAC-------------AGCAGCAACAGCAAC---AATTTTT     862

ENST000006699    1071 ----TTGGTGTTTGTCATTCTCATGAATGTCTTTACACTTACACTACAAA    1116
                          ||||| ||||        ||.||    |||..|.||          
ENSMUST000001     863 TAAATTGGT-TTTG--------ATCAA----TTTTGAGTT----------     889

ENST000006699    1117 TGGATGGACCTCCAAACCATGTGTAGAATTGTTCTGCATGATTTTCAACT    1166
                                       |||.|.||    |.|||||.|||  .||.||||
ENSMUST000001     890 -----------------CATTTATA----TATTCTGTATG--CTTGAACT     916
```

```
ENST000006699   1167 TTAT-TTATATAGTATCATACTTGATATATTTTCCCAAGTTGTTT-----   1210
                     ||.| ||.|||||||         ||.|.|||||.|   ||.|.|
ENSMUST000001    917 TTTTGTTTTATAGTA---------ATTTGTTTTCTC---TTCTCTGGGAC    954

ENST000006699   1211 -TACTTGT-----GAAA--TTCATCCATGT   1232
                      ||| |||     ||||  ||.||..|..|
ENSMUST000001    955 ATAC-TGTAGATGGAAACTTTAATAAAAAT    983
```

Figure S6: Luciferase activity of *Ptchd1* promoter 5’ truncations in stably-transfected monoclonal P19 lines following differentiation, with and without KCl treatment. Luciferase activity for monoclonal stably-transfected P19 lines expressing reporter cassettes with 5’ truncations of the *Ptchd1* promoter ranging from -1782 to +17 relative to the TSS at DAI 16. Data are expressed as the mean ± SEM for each group and were analyzed using a one-way ANOVA followed by a Tukey’s HSD test (**$p < 0.01$ between the indicated groups; $n = 3$ separate clones analyzed for each construct; also, all constructs showed significantly greater expression ($p < 0.01$) relative to the -146 to +17 construct).

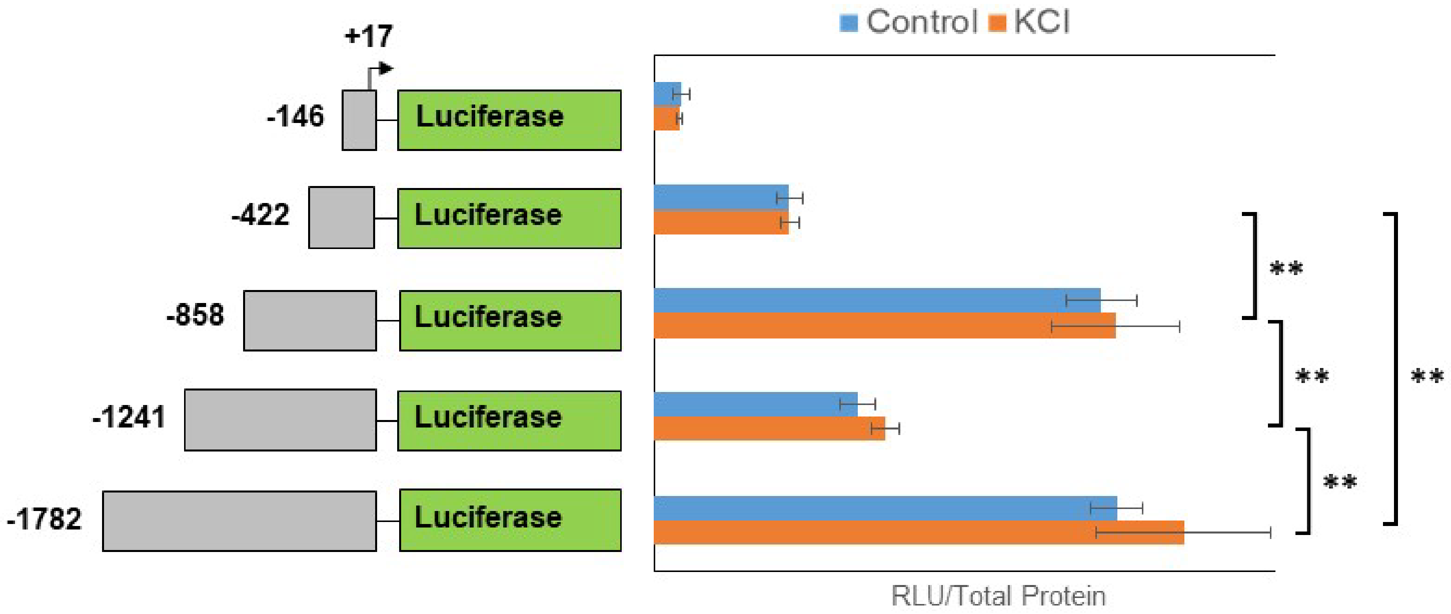

Figure S7. Chromatin accessibility downstream of *Ptchd1* in P0 brain subregions. ENCODE browser images indicating DNase-seq. data in the mouse P0 **A)** forebrain, **B)** midbrain, and **C)** hindbrain. N.B. the differences in scale for the y-axes.

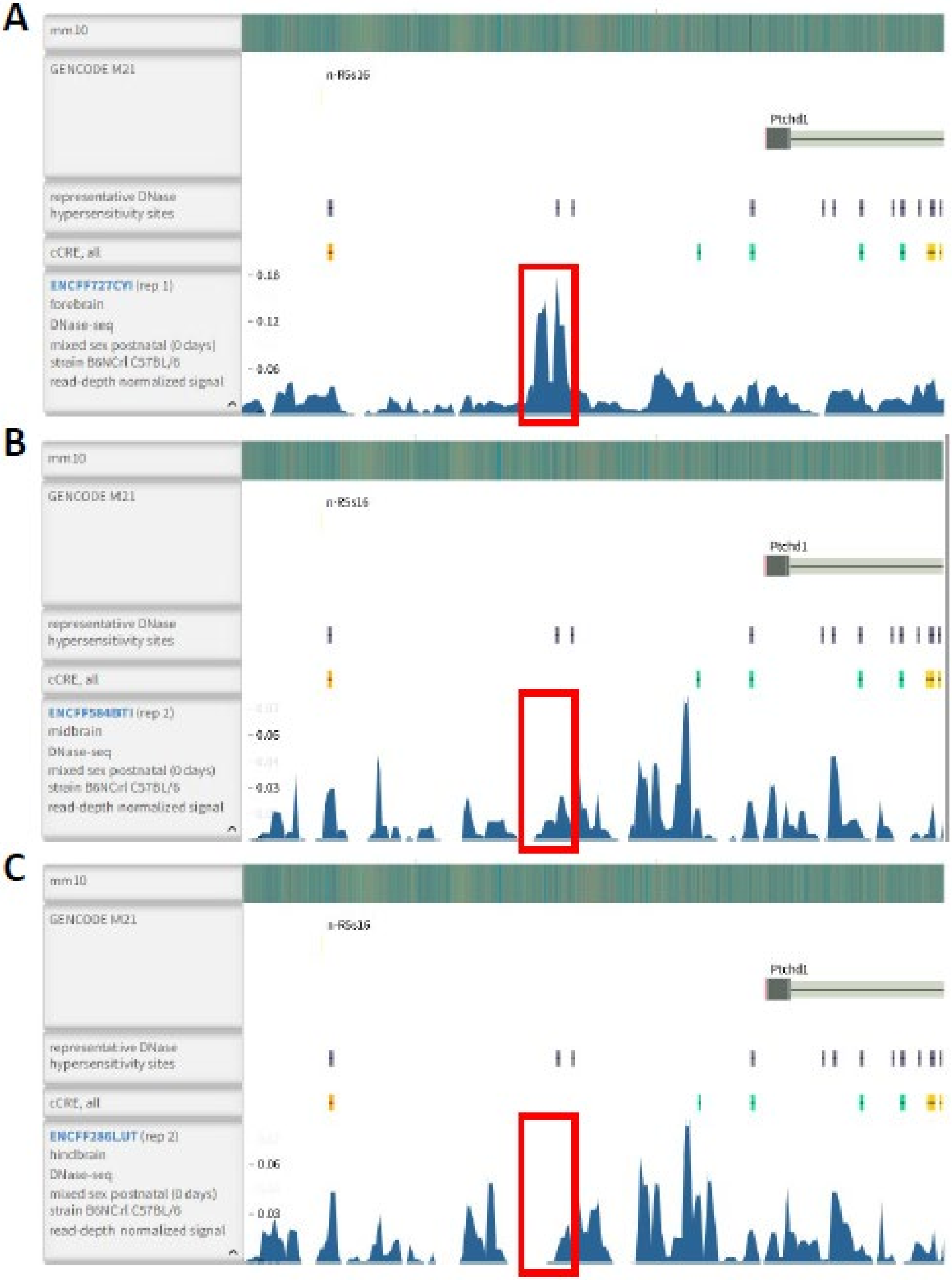

Figure S8. Chromatin accessibility downstream of *Ptchd1* in P0 forebrain and peripheral tissues. N.B. the difference in scale for the y-axes compared to forebrain.

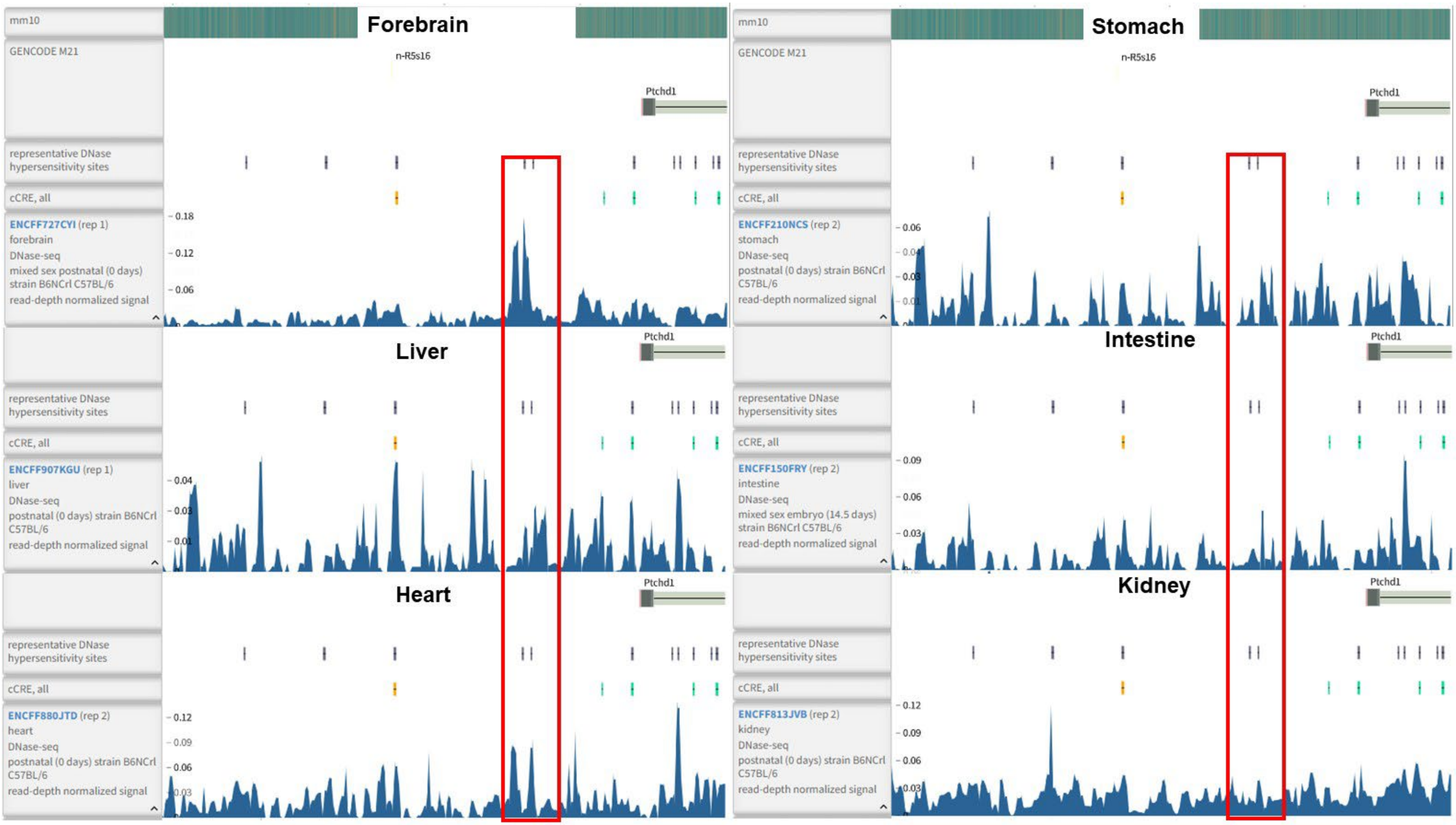

Figure S9. Chromatin accessibility downstream of *PTCHD1*. ENCODE browser images indicating DNase-seq. data in the human embryonic **A)** forebrain and **B)** liver. N.B. the difference in scale for the y-axis in A) brain versus B) liver.

A)

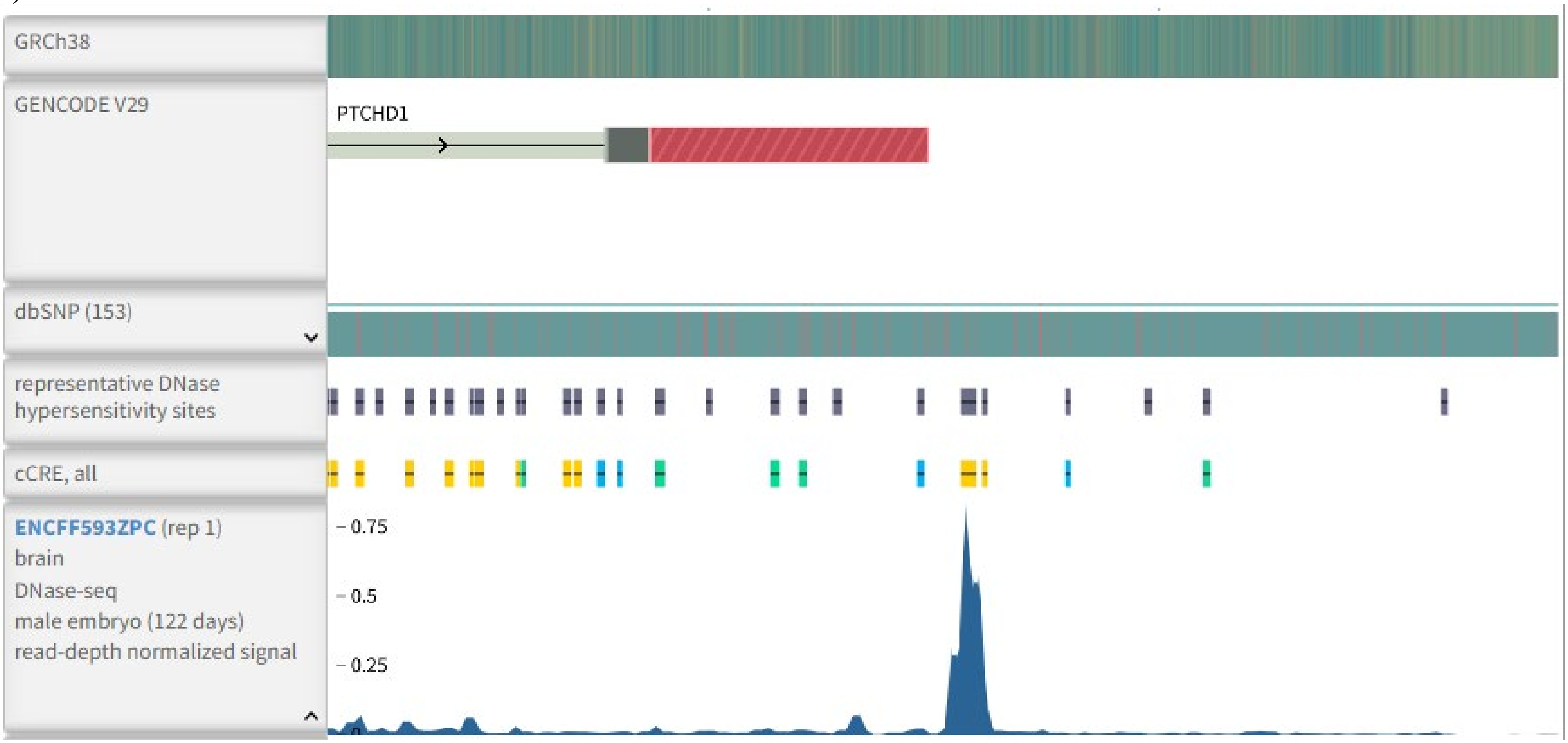

B)

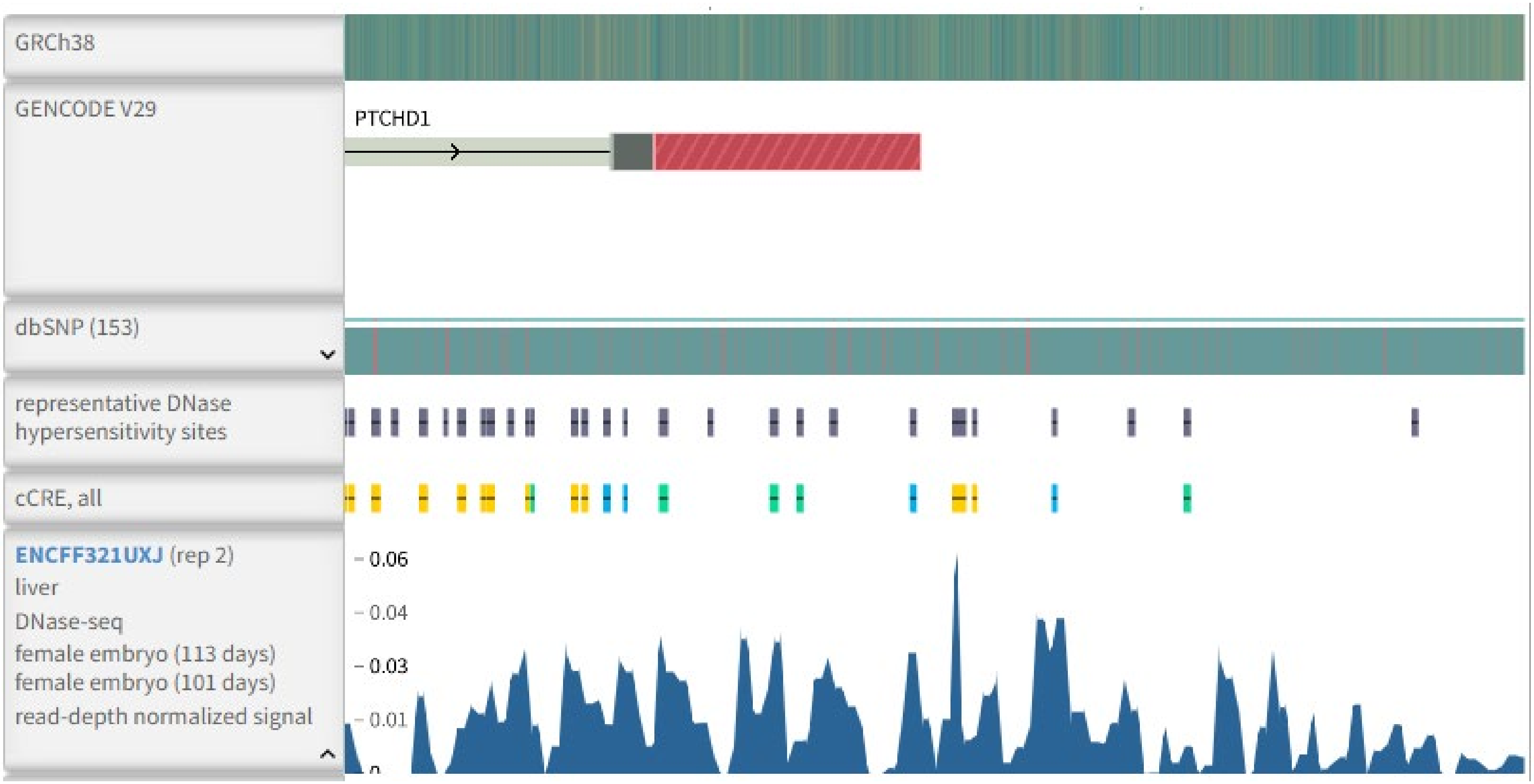
GRCh38
GENCODE V29
PTCHD1
dbSNP (153)
representative DNase hypersensitivity sites
cCRE, all
ENCFF321UXJ (rep 2)
liver
DNase-seq
female embryo (113 days)
female embryo (101 days)
read-depth normalized signal
0.06
0.04
0.03
0.01
0

Figure S10. Conservation of downstream distal enhancer elements in mouse, macaque and human. Genomic conservation of the human UCSC-annotated enhancers **A)** EH38E2746819 and **B)** EH38E2746821.

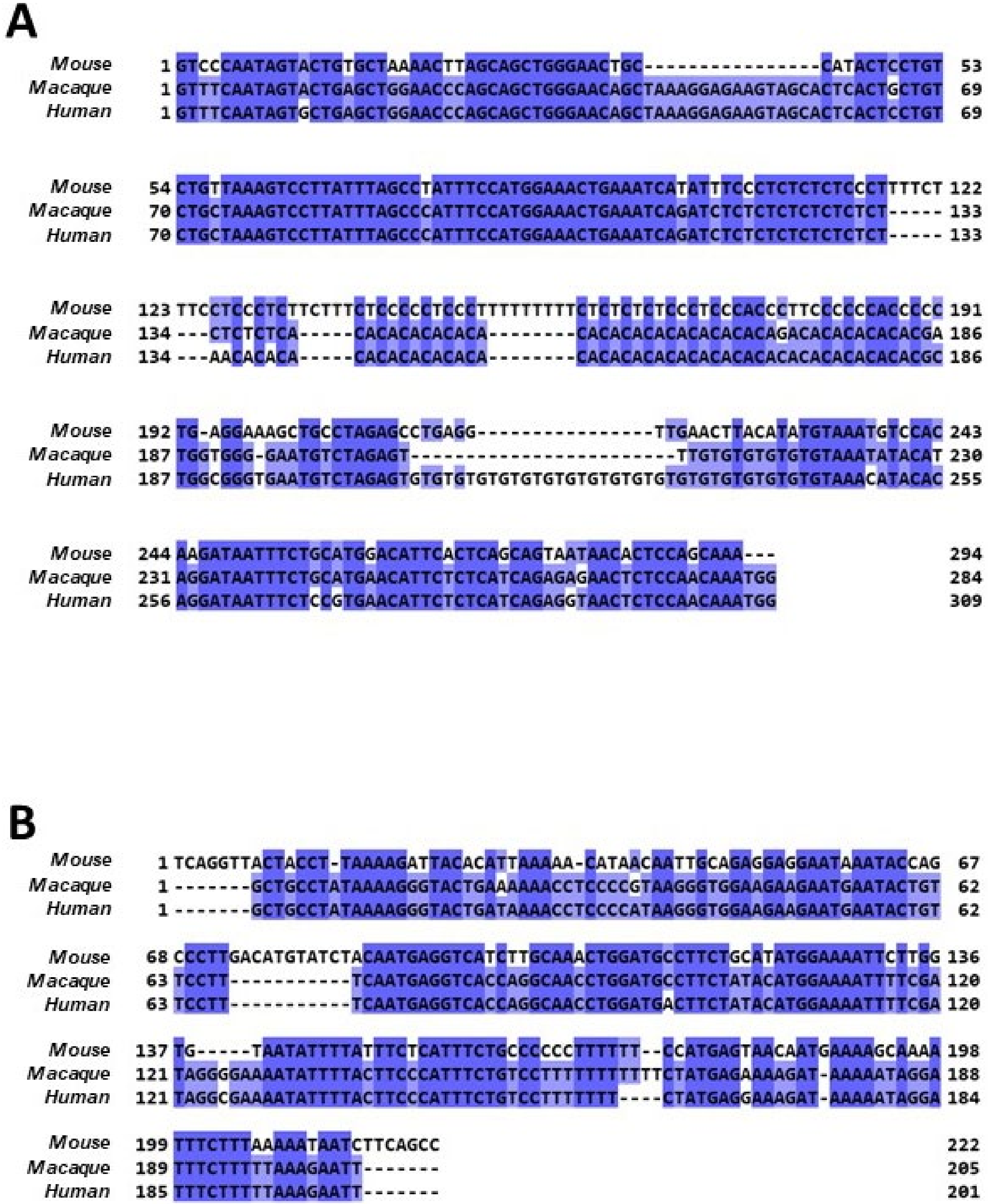

Figure S11. Position-frequency matrices for five conserved putative TFBSs.

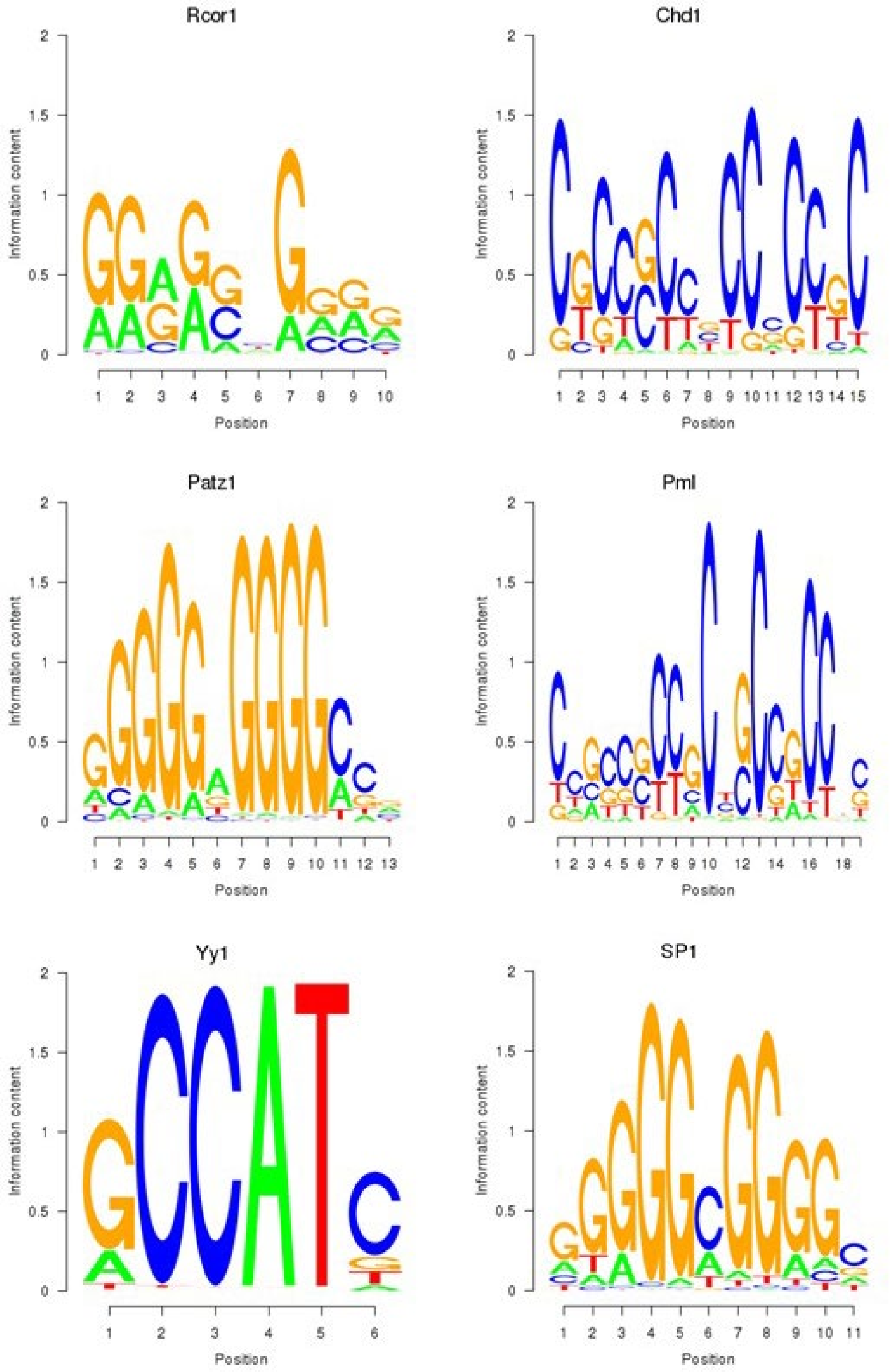

Figure S12. Cruciform DNA element within *PTCHD1* downstream enhancer. A) UCSC genome browser image indicating the location of an inverted repeat sequence within the EH38E2746818 enhancer downstream of the *PTCHD1* 3'UTR, as well as showing sequence conservation across 30 mammals (Multiz alignment). **B)** This inverted repeat sequence is predicted to form a putative cruciform DNA element.

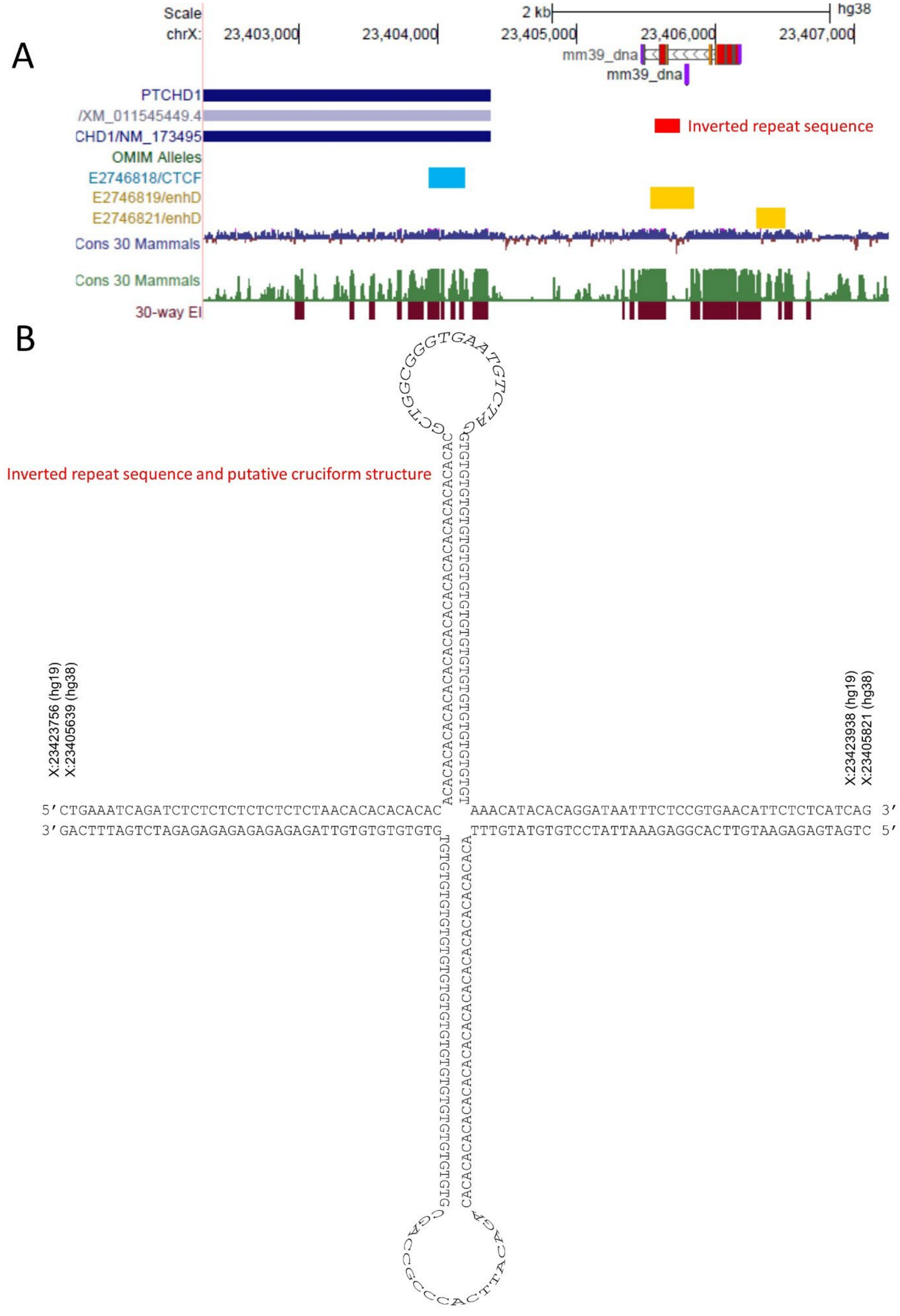

Table S5: MSSNG rare variants (≤0.01) within upstream and downstream regulatory regions (accessed 17 Oct 2023). Hemizygous variants are included (either *de novo* or maternally inherited). Coordinates use hg38. Upstream CTCF regions have been reported in the DECIPHER genome browser (www.deciphergenomics.org) and ensembl.org, flanking the human promoter/enhancer regions (see Fig S11). The downstream enhancer region EH38E2746819, which contains the large palindromic simple tandem repeat sequence appears to be highly polymorphic, and variants in this region are not included in the table. No ClinVar variants were identified within these upstream or downstream regions. * indicates multiplex, male/male families, # indicates multiplex mixed male/female affected families. Excess familial sharing of variants between male affecteds in these regions was not observed.

MSSNG ID	Coordinates	Allelic depth	gnomAD freq	Comments
**Upstream**				
1-0054-004*	23330405G>C	0,34	0	CTCF (ENSR00001158811)
5-0137-003	23330431C>G	0,8	0.00012519	CTCF (ENSR00001158811)
AU3808304*	23330445T>G	0,7	0	CTCF (ENSR00001158811)
1-1167-003	23330479G>C	0,18	0	CTCF (ENSR00001158811)
MSSNG00426-003	23330596C>T	0,13	0.00139845	CTCF (ENSR00001158811)
MSSNG00349-003*	23330596C>T	0,14	0.00139845	CTCF (ENSR00001158811)
MSSNG00349-004*	23330596C>T	0,17	0.00139845	CTCF (ENSR00001158811)
AU2521301	23330596C>T	0,11	0.00139845	CTCF (ENSR00001158811)
MSSNG00043-004A#	23330596C>T	0,9	0.00139845	CTCF (ENSR00001158811)
1-0234-003*	23330596C>T	0,16	0.00139845	CTCF (ENSR00001158811)
1-0234-004*	23330596C>T	0,15	0.00139845	CTCF (ENSR00001158811)
5-0091-003	23330596C>T	0,16	0.00139845	CTCF (ENSR00001158811)
5-5203-003	23330596C>T	0,25	0.00139845	CTCF (ENSR00001158811)
1-1067-003	23333442T>C	0,11	0	CTCF (ENSR00001291355)
5-5015-003*	23333547T>A	0,23	0.00183081	CTCF (ENSR00001291355)
5-5015-005*	23333547T>A	0,20	0.00183081	CTCF (ENSR00001291355)
1-0354-006	23333547T>A	0,16	0.00183081	CTCF (ENSR00001291355)
MT_26.3	23333547T>A	0,20	0.00183081	CTCF (ENSR00001291355)
AU4452302	23333547T>A	0,17	0.00183081	CTCF (ENSR00001291355)
AU2285301	23333547T>A	0,17	0.00183081	CTCF (ENSR00001291355)
MT_160.4*	23333547T>A	0,7	0.00183081	CTCF (ENSR00001291355)
AU054103	23333562G>A	0,22	0.0001973	CTCF (ENSR00001291355)
AU027506	23333569T>G	0,17	7.92E-05	CTCF (ENSR00001291355)
REACH000713*	23333587T>A	0,27	0	CTCF (ENSR00001291355)
REACH000709*	23333587T>A	0,39	0	CTCF (ENSR00001291355)
MSSNG00203-003	23333593T>C	0,14	0	CTCF (ENSR00001291355)
REACH000589	23333732G>A	0,23	0.00233319	RCOR1 binding site
MSSNG00028-004*	23335211C>T NM_173495:c.336C>T :p.Thr112Thr	0,38	0.00037646	CTCF (ENSR00001291356)
7-0320-003	23335265G>T	0,20	0	CTCF (ENSR00001291356)
5-5209-003	23335348C>T	0,24	0.00176047	CTCF (ENSR00001291356)
1-0161-004*	23335348C>T	0,15	0.00176047	CTCF (ENSR00001291356)
1-0161-003*	23335348C>T	0,15	0.00176047	CTCF (ENSR00001291356)
**Downstream**				
AU056003*	23405564G>A	0,8	7.07E-06	Enhancer (EH38E2746819)
1-0079-008*	23405596C>G	0,9	0	Enhancer (EH38E2746819)
1-0161-004*	23406386A>C	0,12	0.00015777	Enhancer (EH38E2746821)
1-0161-003*	23406386A>C	0,16	0.00015777	Enhancer (EH38E2746821)

AU1933302#	23406398T>G	0,21	0.00018225	Enhancer (EH38E2746821)
2-1845-003	23406425C>T	0,12	0	Enhancer (EH38E2746821)
1-0662-003	23406425C>T	0,20	0	Enhancer (EH38E2746821)
1-1154-003	23406450G>T	0,21	0.00262224	Enhancer (EH38E2746821)
1-1116-003	23406450G>T	0,30	0.00262224	Enhancer (EH38E2746821)
AU4024303	23406487T>A	0,23	0.00728119	Enhancer (EH38E2746821)
AU4181304	23406487T>A	0,18	0.00728119	Enhancer (EH38E2746821)
1-0580-003*	23406487T>A	1,16	0.00728119	Enhancer (EH38E2746821)
SJD_23.3	23403960C>T	0,17	8.24E-05	CTCF (EH38E2746818)
AU3639306	23403986G>C	0,18	0.00375158	CTCF (EH38E2746818)
SJD_63.3	23404025C>T	0,22	0	CTCF (EH38E2746818)
MSSNG00111-004*	23404108C>G	0,17	0.00140897	CTCF (EH38E2746818)
MSSNG00111-003*	23404108C>G	0,23	0.00140897	CTCF (EH38E2746818)
1-0289-004#	23404174A>G	0,11	0	CTCF (EH38E2746818)
2-1315-003	23409391T>C	0,22	0	CTCF (EH38E2746822)
1-0304-003	23409417C>G	0,13	1.58E-05	CTCF (EH38E2746822)
1-0190-003	23409458G>A	0,11	0	CTCF (EH38E2746822)
AU3397302	23409490G>A	0,16	0.00582968	CTCF (EH38E2746822)
1-0811-003	23409490G>A	0,23	0.00582968	CTCF (EH38E2746822)
1-0894-003	23409498T>G	0,19	0.00021091	CTCF (EH38E2746822)

Fig S13: UCSC Browser (human, hg38) indicating location of *PTCHD1* upstream putative CTCF regulatory regions.

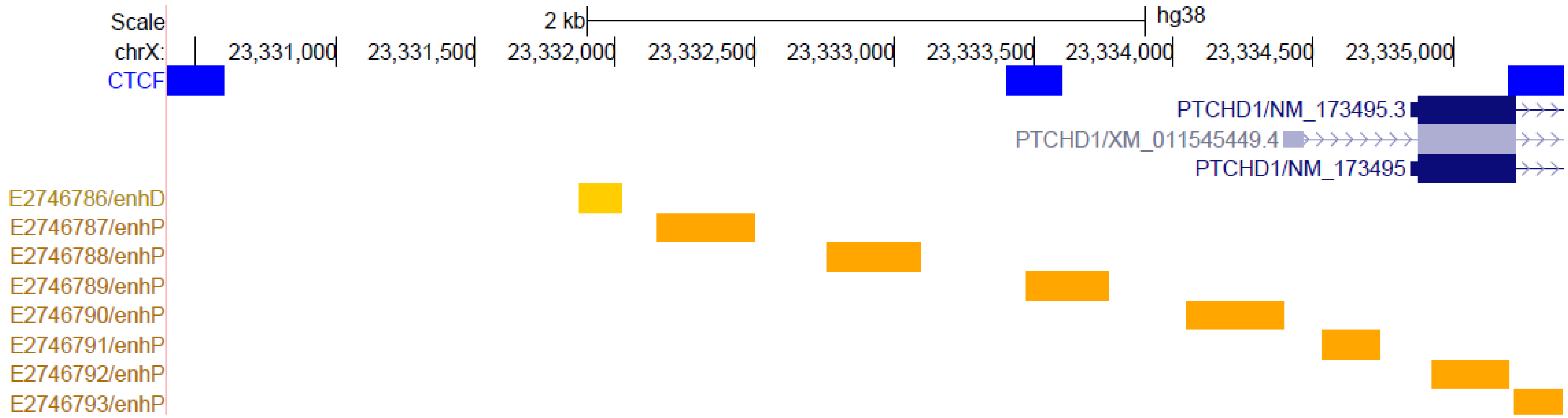

Supplement: Supplementary file 1 — Supplementary Information. [file 41598_2023_46673_MOESM1_ESM.pdf]
